# Comparative ecophysiology of a critically endangered (CR) ectotherm: Implications for conservation management

**DOI:** 10.1371/journal.pone.0182004

**Published:** 2017-08-16

**Authors:** Andrea F. T. Currylow, Angelo Mandimbihasina, Paul Gibbons, Ernest Bekarany, Craig B. Stanford, Edward E. Louis, Daniel E. Crocker

**Affiliations:** 1 Integrative and Evolutionary Biology, University of Southern California, Los Angeles, California, United States of America; 2 Durrell Wildlife Conservation Trust, Antananarivo, Madagascar; 3 Turtle Conservancy, Ojai, California, United States of America; 4 Herpetology Section, Los Angeles County Natural History Museum, Los Angeles, California, United States of America; 5 Madagascar Biodiversity Partnership, Conservation Genetics Department, Omaha’s Henry Doorly Zoo and Aquarium, Omaha, Nebraska, United States of America; 6 Department of Biology, Sonoma State University, Rohnert Park, California, United States of America; University of Sao Paulo, BRAZIL

## Abstract

Captive breeding is a vital conservation tool for many endangered species programs. It is often a last resort when wild animal population numbers drop to below critical minimums for natural reproduction. However, critical ecophysiological information of wild counterparts may not be well documented or understood, leading to years of minimal breeding successes. We collected endocrine and associated ecological data on a critically endangered ectotherm concurrently in the wild and in captivity over several years. We tracked plasma concentrations of steroid stress and reproductive hormones, body condition, activity, and environmental parameters in three populations (one wild and two geographically distinct captive) of ploughshare tortoise (*Astrochelys yniphora*). Hormone profiles along with environmental and behavioral data are presented and compared. We show that animals have particular seasonal environmental requirements that can affect annual reproduction, captivity affects reproductive state, and sociality may be required at certain times of the year for breeding to be successful. Our data suggest that changes in climatic conditions experienced by individuals, either due to decades-long shifts or hemispheric differences when translocated from their native range, can stifle breeding success for several years while the animals physiologically acclimatize. We also found that captivity affects stress (plasma corticosterone) and body condition of adults and juveniles differently and seasonally. Our results indicate that phenotypic plasticity in reproduction and behavior is related to environmental cues in long-lived ectotherms, and detailed ecophysiological data should be used when establishing and improving captive husbandry conditions for conservation breeding programs. Further, considering the recent revelation of this tortoises’ possible extirpation from the wild, these data are critically opportune and may be key to the survival of this species.

## Introduction

Assessing the relative extinction risk of a species depends on the population size, genetic diversity, distribution, demographics, stressors, life history traits, and threats, amid other criteria. These risks are evaluated by the International Union for Conservation of Nature (IUCN), which publishes conservation statuses in the IUCN Red List to highlight those species at higher risk (http://www.iucnredlist.org). The IUCN Red List defines a taxon as Critically Endangered (CR) when data indicates that the species is facing an extremely high risk of extinction in the wild defined by a high percentage and recent population size reduction, a highly restricted geographic range, a very low adult population size estimate, or a high probability of extinction in the wild within 10 years. This designation is superseded by Extinct in the Wild (EW) if extensive surveys fail to record any animal of a species within its expected range or habitat. It may be only a few combined factors that cause a CR species to become EW; the risk of extinction increases and the recovery efforts become more challenging if we lack natural ecological information [1]. Conservation strategies for CR wildlife involves the captive breeding of wild-caught individuals for enhancing the captive gene pool and repatriation to native habitats, in what have come to be known as assurance colonies. As with all conservation management plans, the captive breeding approach is accompanied by a variety of challenges [1]. Successful relocation, including to captivity, is dependent upon knowledge of biological constraints (e.g., natural environmental and habitat requirements) as well as basic ecology and health parameters [1–5]; information that is often unavailable in emergency recovery situations [6]. In addition, animals can become stressed in captivity, making reproduction physiologically impossible [7–9]. There are successes, however; animal populations reduced to functionally extinct numbers have been taken from the wild into captivity and, over time, brought into reproductive condition such as the California condor and black-footed ferret. Those successes came through multiple studies on reproduction, mirroring wild conditions, social behavior, and more [10–13]. The captive breeding and eventual repatriation of these individuals and their offspring to native habitats was arguably the single most important step for the survival of the species [1].

Creating and maintaining captive husbandry conditions that promote natural behaviors, health, and reproductively viable individuals is challenging, particularly when little ecological information exists for rare/elusive taxa. These challenges are confounded by the protracted survival probability curve in long-lived species [14]. This is pertinent with CR species in particular, as effective captive breeding colonies may one day act as source populations for extirpated wild ones (i.e., assurance colonies). Understanding typical seasonal environmental and physiological patterns in natural populations (i.e., baseline) should be the standard to which we compare captive populations. We can use baseline variation of body conditions (natural seasonal fluctuation in size and weight) and temporal changes of hormone concentrations, activity, and environmental fluctuations recorded in wild populations to evaluate health of captive populations because these factors relate to aspects of stress and reproduction (e.g., immune function, energy budgets, behavior, etc.; [15–17].

Stress is a physiological response to a perceived threat. It can greatly affect an animal’s ability to cope with changes in environment. Though adaptive in the short term, the stress response can disrupt normal, seasonal processes such as reproduction [18, 19]. When a perceived stressor occurs, the animal’s hypothalamus-pituitary-adrenal (HPA) axis is stimulated and begins to produce a cascade of hormones (catecholamines and glucocorticoids), triggering immediate behavioral and physiological responses intended to promote survival of the organism [20, 21]. When a stressor persists over longer periods of time (chronic stress), the HPA continues producing the glucocorticoid stress hormone, which can have far-reaching deleterious effects on diel and seasonal functions such as immune response and reproduction [22–25]. Though the primary glucocorticoid stress hormone (CORT) in most mammals and fish is cortisol, it is corticosterone in reptiles and birds [26]. The production of CORT can help the animal divert physiological resources from non-vital functions to those which will aid in short-term survival ([27]. An increase in the production of CORT elevates blood glucose concentrations and constrains the production on gonadal steroid secretions, growth, and immune system function as well as causes altered behaviors [19, 20, 28]. Continued production of CORT above baseline values (baseline = unstressed concentrations; [29] throughout a season and/or year(s) (i.e., chronic stress) may therefore prevent an animal from entering a reproductive state. For wild animals, particularly for those CR species under threat of extinction, the inability to reproduce in any given year might be the difference between population recovery and extirpation.

Animals may perceive various types of stressors depending on their surroundings and situation. In the wild, stressors may be in the form of environmental perturbations (e.g., extreme temperatures, drought, strong storms, etc.), human-wildlife conflict (e.g., encroaching agriculture, habitat harvesting, grazing, etc.), inter- and intraspecific interaction (e.g., invasive species or competition), or simply a lack of resources (e.g., cover, forage, mates, etc.). Altered activity patterns can be exhibited by stressed individuals, such as increased wandering behaviors in search of forage or mates, causing them to divert physiological resources from functions such as reproduction to survival [19]. In captivity, activity and physiology may be altered due to stressors such as insufficient space, inadequate nutrition, limited environmental enrichment, continual human perturbation, etc. [30]. Through the investigation of stress and reproductive hormones, we can determine the reproductive state of individuals and interpret seasonal hormone profiles to characterize populations.

Reproductive rates and patterns are used as indicators of biological fitness [31] and can be tracked or monitored physically and hormonally. Stress and reproductive state are exhibited by an animal through physical signs such as altered body condition and/or activity patterns [19]. Clinically healthy and reproducing animals are generally described as being in better body condition (larger and heavier) and exhibiting normal activity (movement patterns and behaviors; [31–33]). Defining and measuring clear categories of these indicators as well as associated environmental parameters in wild animal populations helps define baseline physical and ecological data of a particular species. Comparisons of endocrine hormone profiles along with environmental and behavioral data from wild populations will provide baseline values on a suite of ecophysiological parameters required for successful management.

Of particular concern are CR reptiles because an ectotherm’s behavior and physiology are obligatorily tied to environmental conditions; even relatively minor environmental perturbations could have ongoing effects [34–37]. These perturbations may cause years of altered behavior and health, making husbandry particularly difficult with these taxa. Temperature has been shown to be the most important cue for reproduction in reptiles [38], so fluctuations in environmental temperatures can disrupt reproduction. Captive breeding and reintroductions of ectotherms can be successful, but past efforts emphasize the need for detailed analyses of multiple environmental factors alongside reproduction and health monitoring of animals themselves [39, 40]. When research science and captive management work together, key features which sustain healthy, wild populations are identified and implemented in captivity [41]. Research needs to be conducted to assess the key environmental, ecological, and physiological parameters for ectotherms [42], and a call has been made for habitat features to be closely evaluated for their importance in captive management situations [43].

**Fig 1:**
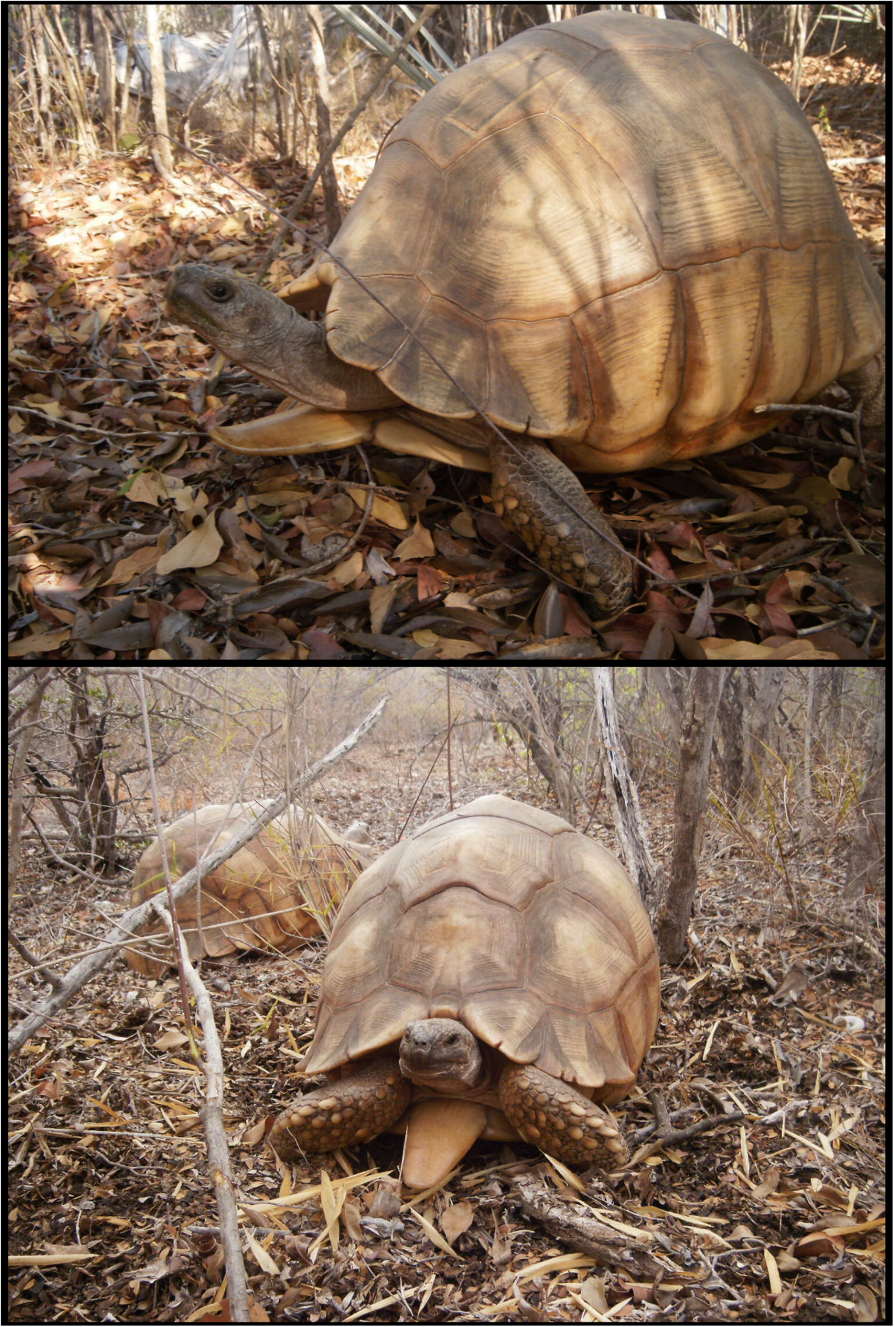
Wild *Astrochelys yniphora* in its native habitats. This “golden tortoise” is near extinct in the wild (EW) due to wildlife smugglers.

We investigated the ecophysiology of a CR ectotherm from a biodiversity hotspot, Madagascar. All endemic Malagasy chelonians are threatened by extinction [44], and Madagascar’s ploughshare tortoise (*Astrochelys yniphora*) is widely considered the most endangered tortoise species in the world [45–47]. This is Madagascar’s largest chelonian species [48] with a highly-domed shell, extended epiplastral projection, a unique golden color, (Fig 1) and a highly restricted in range [49–51]. Until very recently, population estimates of this species hovered around 400 [50, 52–54], but since January 2016, this species is close to extirpation at all four of their historically wild locations [55]. Previous declines were attributed to various threats including brush fires, bush pigs, habitat loss, and collection for pets [44, 46, 56, 57]. But it is the very recent and dramatic increase in collection for the illicit pet trade of this “golden tortoise” that is causing the present precipitous drop; perhaps already falling into an EW designation [55, 58, 59].

Due to past and ongoing threats to the species, the development of captive assurance colonies is being considered, but successful reproduction over multiple generations has thus far only occurred in one captive collection, which is very near to the species’ native range. Little is known about reproductive state and seasonality in wild *A*. *yniphora* [60], though behavioral studies have documented mating and nesting activities [50, 61]. Understanding the reproductive physiological changes that wild ectotherms undergo, along with those of the successfully reproducing captive animals, will give conservation managers a baseline from which to gauge successes and challenges. Using *A*. *yniphora* as a model, we aimed to: 1) characterize baseline and seasonal patterns of stress and reproductive hormones in a CR ectotherm and use those characterizations to test; 2) if those characterizations relate to body condition and activity; 3) whether captivity affects stress, reproduction, and body condition of animals; and 4) which key environmental factors influence herpetofauna health and behaviors for use in captive management.

## Materials and methods

Endocrine control of reproductive cycles in ectotherms is generally documented using monthly mean hormone concentrations, which ebb and flow, peaking multiple times a year for species that nest multiply [62]. To characterize baseline and variation in reproductive and stress hormones of *A*. *yniphora*, we conducted surveys for, and sampled animals from, both wild and captive populations throughout three years (2013–2015).

### Animals, populations, and sampling

As part of another study, we conducted distance and meandering transect surveys in four areas where remaining natural and wild populations of *A*. *yniphora* are known to occur [45, 49, 51] from early 2013 through late 2015. We sampled animals opportunistically during surveys and tracking. Sampling occurred during both the animals’ natural active seasons (wet season; November–April) and inactive seasons (dry season; May–October) during 2013–2015. Courtship, mating, and nesting are seasonal and generally occur from December through May. Upon detection, we first collected a blood sample, animal weight (g) using spring scales, maximum carapace length (MCL; mm) using calipers, visually sexed using secondary sexual characteristics (as described in [48]), observed activity prior to disturbance (walking, resting in open, resting in vegetation, eating, basking, or other), and environmental variables (air and ground temperatures, ground humidity, cloud cover, and vegetation cover) as described in [63]. Upon capture, each animal was individually PIT tagged and uniquely marked using a fine saw blade to create notches along the marginal scutes following a modified Cagle scheme [64–66]. Tracked animals were identified by those identifiers and associated transmitter frequencies.

Two captive populations (Madagascar and USA) were sampled for comparison to wild animals. The Malagasy population is located approximately 160 km from the native range in northwestern Madagascar, managed by Durrell Wildlife Conservation Trust and is the only known successfully breeding captive population of *A*. *yniphora* in the world [61, 67]. The much smaller U.S. population in the opposing global hemisphere was included as an outgroup for comparison. The U.S. population is housed at the Behler Chelonian Center in southern California, is managed by Turtle Conservancy, and began recording courtship and nesting activities within the group in November 2013. Details on the captive husbandry of these facilities were not recorded for the purposes of this study. We sampled these populations monthly whenever possible from 2013 through 2015. We included 16 males, 21 females, and 42 sub-adults from the Madagascar captive population and the single male, the two females, and 10 sub-adults from the U.S. captive population. In both populations, individuals were frequently paired for mating and monitored by staff. Pairings between adult individuals were recorded.

We collected blood samples (~1 cc) from the subcarapacial sinus [68] using a 23-gauge needle on a 3-cc syringe. This method calls for minimal restraint and does not require head or limb extraction, which reduces distress during sampling [69]. Blood samples that were collected at facilities with electricity were processed following the procedure described in [70]. We modified this procedure when in the field in remote Madagascar (see details in [71]). Briefly, we placed wild animal samples into heparinized vials and onto cool packs in an insulated bag while completing surveys and hiking back to camp (generally within 5 hours), where we field centrifuged samples using solar-stored power, pipetted plasma to a cryovial, and immediately submerged tubes in liquid nitrogen for field storage until transfer to a -20° freezer (generally within 1–4 weeks; [71]). Samples were imported to the U.S. on dry ice and all samples were transferred to a -80°C cryogenic freezer at the laboratory. All animals were handled per the USC Institutional Care and Use Committee Protocol #12046. Passes and permits for this work were acquired through Madagascar National Parks and the Ministry of Environment, Ecology, and Forests. (permit #s 008/13, 009/13, 214/13, 271/13, 112/14, 129/14, 005/15, 006/15, and 035/16). Samples were exported from Madagascar and imported to the USA under permit from the Convention on International Trade in Endangered Species of Wild Fauna and Flora (CITES) (export permit #118C-EA03/MG15, import permit #14US34804B/9).

### Enzyme-linked immunosorbent assays

To understand natural variation in stress and give us the ability to compare with captive populations, we quantified plasma corticosterone (CORT) using enzyme immunoassays (Cayman Chemical, Ann Arbor, MI, USA; kit #500655) as in [71]. All samples were run in duplicate or triplicate and any pair with coefficient of variation (CV) greater than 15% were re-analyzed. Inter- and intra-assay CVs were 5% or less. The assay platform was validated for use in *A*. *yniphora*. Serial dilutions of pooled plasma yielded curves that were parallel to the standard curve (ANCOVA, *P* > 0.05). Accuracy was assessed using recovery of known standard additions to pooled plasma (*r*^2^ = 0.98; mean recovery = 94.3 ± 4.2%).

### Radioimmunoassays

**Fig 2:**
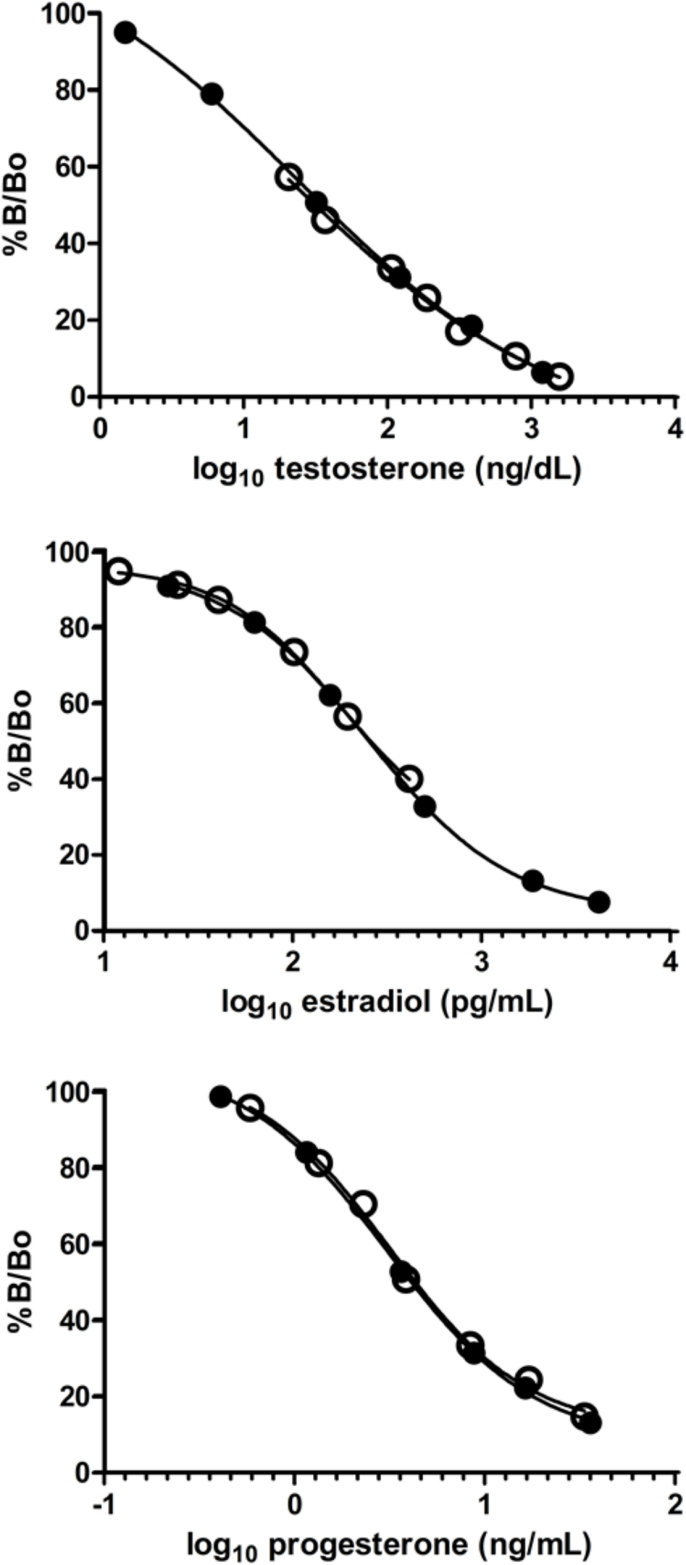
Radioimmunoassay hormone validation curves for *Astrochelys yniphora*. Closed circles are standards, open circles are serial diluted pooled plasma samples.

To gain a clear picture of reproductive cycles and potential in each population, we measured plasma concentrations of Testosterone (T), estradiol-17β (E2), and progesterone (P) using commercially available radioimmunoassay kits (Immuno-Biological Laboratories Inc., Minneapolis, MN. #KIR1709 [T], #KIP0629 [E2], and #KIP1458 [P]). Kits were validated using pooled plasma samples to perform serial dilutions compared against standard curves. Dilutions displayed parallelism to the standard curves, indicating that binding affinities were similar in samples and kit standards (Fig 2). Recovery of added standards averaged 96.2 ± 2.3%, 102.3 ± 3.2%, 98.3± 3.1% for T, E2, & P respectively. Each sample was run in duplicate and inter-assay variation averaged 1.8–5.1%. Assay sensitivities were 0.05 ng/mL (T), 2.7 pg/mL (E2), and 0.05 ng/mL (P).

### Statistical analyses

Body condition scores (BCSs) were determined by regressing all records of body weight by their corresponding MCL of each animal, fitting a second degree polynomial curve to the point spread (*r*^2^ = 0.91; e.g., [48]), and calculating the residuals of each point from that line. Negative values represent animals with body conditions below the line (fitted mean). To evaluate seasonal body condition, stress, and reproductive hormone concentrations, we grouped plasma hormone concentrations by population, sex, and/or month. We used linear mixed models (LMM) with animal ID as the repeated measure to test for differences within or between the groupings. When significant differences were present we performed post-hoc comparisons of groups using Tukey HSD. Model residuals were visually assessed for approximate normality and homoscedascity. All analyses were performed in JMP statistical software [72] and significance was determined at *P* ≤ 0.05.

## Results

Due to the small sample sizes from the outgroup (the U.S. captive population), we did not include those data in many of the statistical comparisons. We do include the mean values, however, alongside the Madagascar populations in the figures for visual comparisons.

### Stress hormones

Over the three years of study, we collected 409 samples from 153 individuals that were usable for CORT analyses. Of those, 141 (61♂, 64♀, 16SA) were from wild *A*. *yniphora*, 212 (78♂, 87♀, 47SA) from the Madagascar captive population, and 56 (13♂, 27♀, 16SA) from the U.S. captive population. The median time to blood from first disturbance of the animals was 1.5 min (mean = 2.3 ± SE = 0. 27).

**Fig 3:**
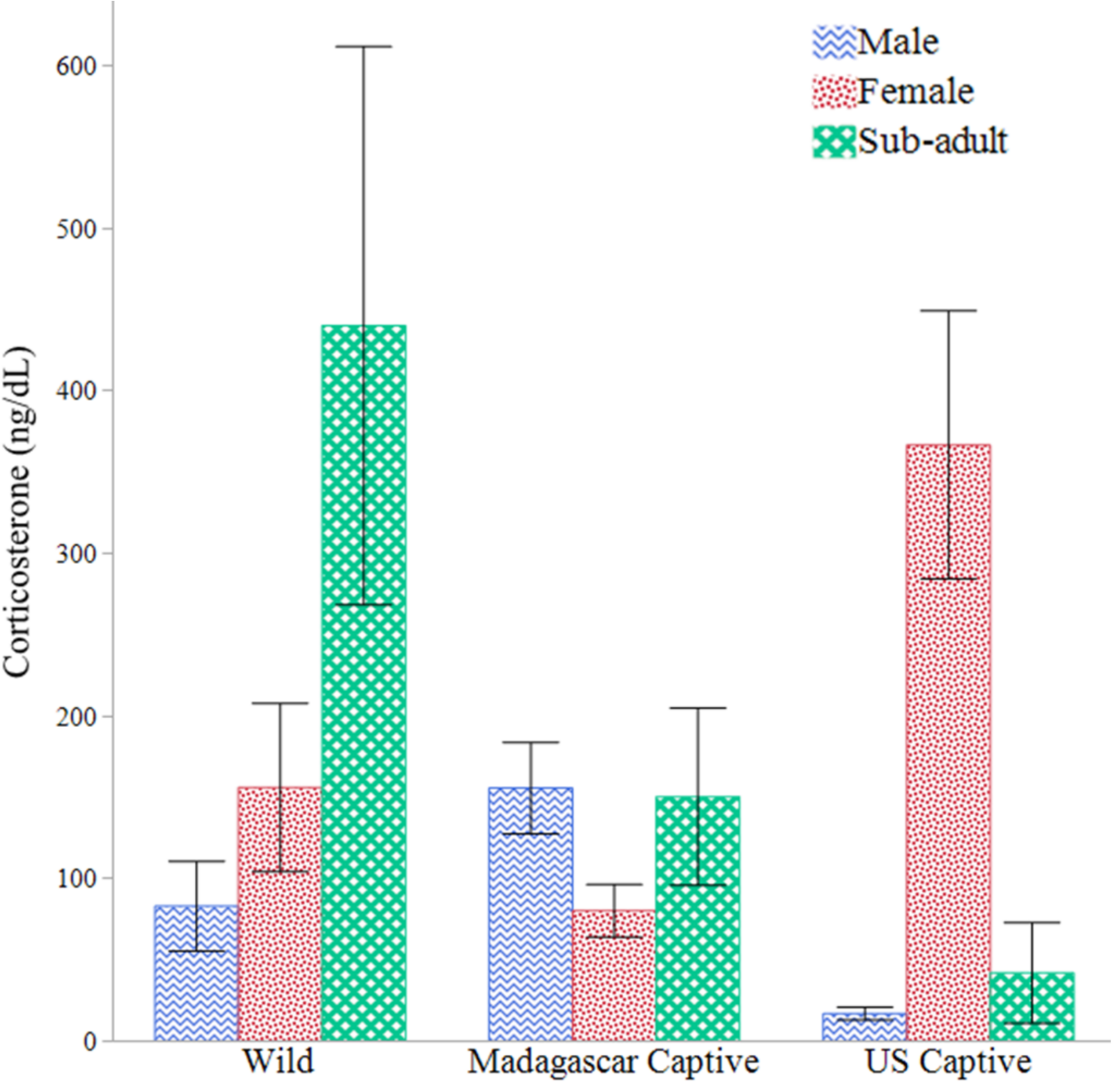
Stress hormones in *Astrochelys yniphora* populations. Summary of mean circulating corticosterone concentrations in male (M), female (F), and sub-adult (SA) *A*. *yniphora* from the wild, a captive population near native range (Madagascar Captive), and a small outgroup, captive population in the U.S from 2013–2015. Error bars = 1SE.

We found no difference in overall CORT concentrations between the populations, months, or sexes (mean ♂ = 90.8 ± 55.4 ng/dL; ♀ = 144.6 ± 50.1 ng/dL; SA = 164.2 ± 48.1 ng/dL. We did note wide variation within the groups, however (Fig 3). When we compared the populations separately, wild sub-adults exhibited significantly higher CORT than either wild adult sex in October (*F*_2,21_ = 5.214, *P* = 0.0145), December (*F*_2,12_ = 16.090, *P* = 0.0004) and May (*F*_2,7_ = 106.435, *P* < 0.0001), and higher than males but not females in January (*F*_2,9_ = 4.435, *P* = 0.0457). Those same wild sub-adult values also had significantly higher CORT than either captive population of sub-adults in January (*F*_2,6_ = 25.569, *P* = 0.0012), February (*F*_2,9_ = 4.435, *P* = 0.0457) and May (*F*_1,8_ = 130.404, *P* < 0.0001), as seen in the higher overall mean.

### Sub-adult hormones

We collected 79 useable plasma samples from sub-adult *A*. *yniphora* within the various populations (16 wild, 47 Madagascar captive, and 16 U.S. captive representing eight, 42, and nine individuals, respectively) over the three years of sampling. Sub-adult *A*. *yniphora* were not targeted for analyses of female sex hormones in this study; therefore, we present limited E2 and P concentrations by month of collection for general reference only.

**Fig 4:**
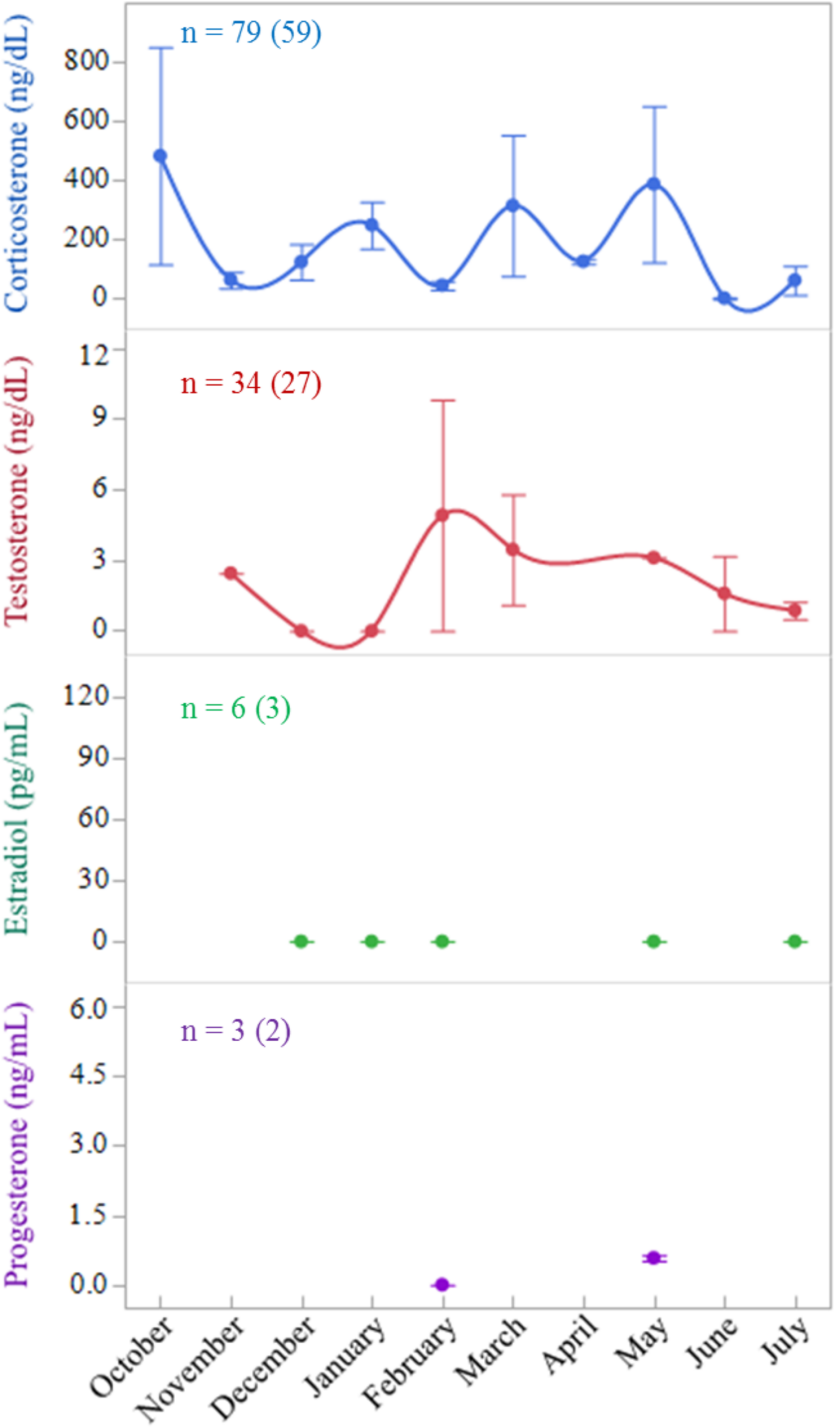
Hormone concentrations in sub-adult *Astrochelys yniphora* populations. Mean monthly steroid hormone concentrations in sub-adult *A*. *yniphora* from all sample populations combined. Smoothing lines included for ease of interpretation, however, no differences between months were significant. Number of samples collected is listed with the number of individuals represented in parentheticals. Error bars represent 1 SE.

We found no differences in hormone concentrations among the sub-adult *A*. *yniphora* in any of the three populations or between the months; therefore, we present the hormone concentrations in a single figure (Fig 4). However, we did detect significant differences in both CORT and T between sub-adults and adult males from the wild population (*F*_1,36_ = 5.466, *P* = 0.0251 and *F*_1,62_ = 4.070, *P* = 0.0480, respectively). For all populations combined, the difference in T remained where adult males exhibited higher T (447.1 ng/dL, SE = 43.2) than sub-adults (*F*_1,115_ = 23.509, *P* < 0.0001). Plasma T in sub-adults averaged 2.8 ng/dL (SE = 1.3). Where there were enough adult female samples to compare (Madagascar captives), we found there to be no differences between adult female and sub-adult T, but there remained a difference between sub-adult and adult male T (*F*_1,89_ = 6.913, *P* = 0.0101). Twenty-two of the 34 sub-adult samples run for T had concentrations below the detection limit of the assay (< 1.5 ng/dL). Sub-adult plasma CORT concentrations averaged 187.9 ng/dL (SE = 49.5). All samples run for E2 (n = 6) returned values below the detectable limit of the assay (< 2.7 pg/mL), and the three samples run for P returned values of 0.00 ng/mL, 0.52 ng/mL, and 0.64 ng/mL.

### Reproductive hormone cycles

In total, we collected 330 usable plasma samples (152♂, 178♀) from adult wild and captive *A*. *yniphora*. Of those, we collected 125 samples (61♂, 64♀) from wild ploughshares in 2013 and 2015, representing 54 individuals (26♂, 28♀); 165 samples (78♂, 87♀) from captive ploughshares representing 39 individuals (17♂, 22♀) housed near their native range in Madagascar between 2013 and 2015; and 40 samples (13♂, 27♀) from the single adult male and the two adult female ploughshares housed in California, USA throughout the course of the study (2013, 2014, and 2015).

### Male hormone cycles

Except where otherwise noted in the following analyses, we excluded the U.S. population because it includes only a single animal and associated risk of pseudoreplication. However, we include the U.S. population’s male in the figures for visual comparison and interpretation of trends.

**Fig 5:**
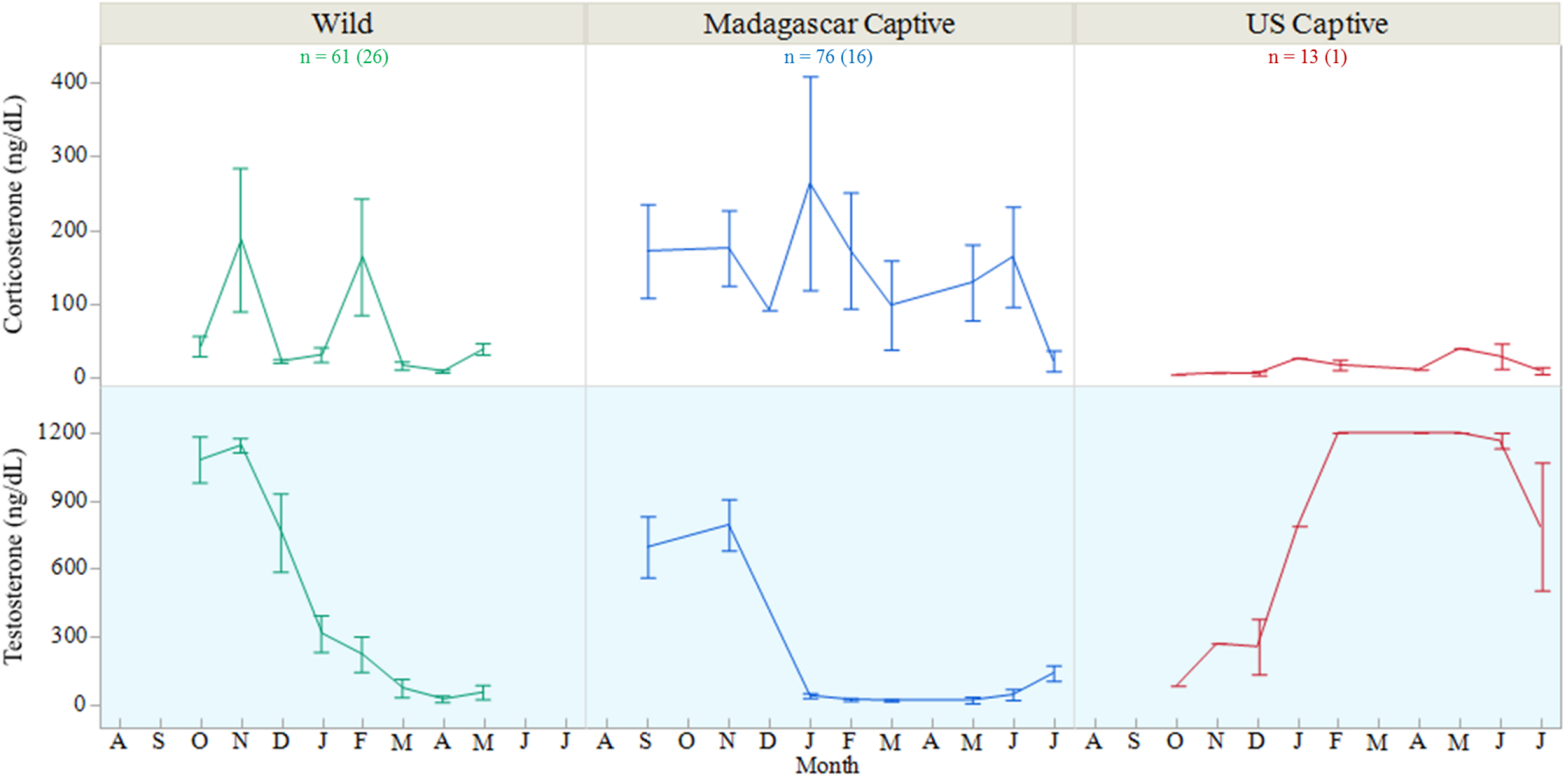
Hormone concentrations in male *Astrochelys yniphora* populations. Mean monthly steroid hormone concentrations in adult male *A*. *yniphora* from three sample populations (wild, Madagascar captive, and U.S. captive), sampled from 2013 through 2015. Number of samples collected from each population is listed with the number of individuals represented in parentheticals. Error bars represent 1 SE.

We found no significant difference in CORT concentrations among male populations (mean range = 74.6–169.0 ± 59.2–63.1 ng/dL) or overall across months (mean range = 80.0–194.9 ± 52.8–93.6ng/dL). However, we did find differences between the populations during December (*F*_1,7_ = 88.033, *P* < 0.0001) and individuals housed in captivity in Madagascar show consistently higher CORT than the other populations (Fig 5). This suggests that a few individuals from the Madagascar captive population account for the wide variation seen within that group. Further, captive males (for which matings were observed) which exhibited higher basal CORT levels were more often those which had sired nests than those males who were not attributed as sires.

Testosterone varied across months (*F*_10,106_ = 45.618, *P* < 0.0001) and between populations (*F*_1,48_ = 12.336, *P* = 0.0010) of male *A*. *yniphora* (Fig 5). The months that all males averaged the highest T were from September through November (802.6–985.1 ± 45.2–73.7ng/dL) while the lowest concentrations occurred in April and May (27.7–68.7 ± 60.4–94.8 ng/dL). When comparing just the two captive populations, we found that males housed in Madagascar had significantly lower T (86.5 ± 53.3 ng/dL) than the male in the U.S. population (884.2 ± 39.7 ng/dL; *F*_1,3_ = 129.340, *P* = 0.0012), and that T varied by month (*F*_1,68_ = 10.6, *P* < 0.0001) with September and November being significantly higher than all other months but April. The captive male of the U.S. population exhibited T concentrations which topped out the detection limit of the assay (1,200 ng/dL) in February and April through June. Six Madagascar captive male samples also topped out the T assay in September and November, while 22 wild male samples did in October, November, and into December. Among those Madagascar captive males for which matings were detected and sires of nests attributed, individuals which were seen to mate in a particular month had significantly lower T (9.1 ± 103.9 ng/dL) than the non-breeders (262.1 ± 45.7 ng/dL) at that time, yet had higher overall mean T during the sample years (186.8 ng/dL vs. 84.4 ng/dL; *F*_1,73_ = 5.217, *P* = 0.0253).

### Female hormone cycles

**Fig 6:**
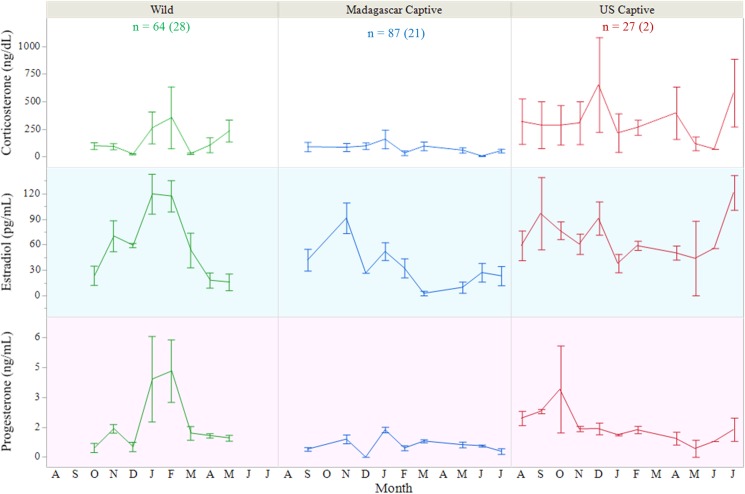
Hormone concentrations in female *Astrochelys yniphora* populations. Mean monthly steroid hormone concentrations in adult female *A*. *yniphora* from three sample populations (wild, Madagascar captive, and U.S. captive), collected in 2013 through 2015. Number of samples collected from each population is listed with the number of individuals represented in parentheticals. Error bars represent 1 SE.

We found there to be no differences in CORT by month or overall within the three populations of female *A*. *yniphora*, yet clear trends are evident (Fig 6) suggesting that individuals account for the variation in the data. When looking at only the captive populations, there was a significant difference in CORT where females housed in the U.S. had significantly higher CORT (376.1 ± 106.2 ng/dL) than those housed in Madagascar (87.3 ± 37.2 ng/dL; *F*_1,15_ = 6.594, *P* = 0.0213). Females from the U.S. captive population also had more variable CORT concentrations by month, the Madagascar captives had consistently low CORT, and the wild animals appeared to peak mid-active season (January-February; Fig 6).

Estradiol varied in females both by month (*F*_11,138_ = 4.270, *P* < 0.0001) and by population (*F*_2,14_ = 9.030, *P* = 0.0032). Overall, females in the Madagascar captive population had lower E2 averages (25.7 ± 6.9 pg/mL) than those in either the wild (62.7 ± 7.4 pg/mL) or U.S. captive (68.6 ± 12.0 pg/mL) populations. All populations varied in the annual pattern of E2, but was highest overall in January and November (81.5–82.2 ± 9.7–11.1 pg/mL) and lowest in April (14.7 ± 15.8 pg/mL). Estradiol exhibited two peaks in the Madagascar populations, but to differing degrees (Fig 6). The U.S. population did not exhibit any distinguishable peaks of E2. Among the two captive populations where nesting had been observed, those females which had been seen to nest within the sample time period (2012–2015) also exhibited higher overall mean E2 concentrations than those which were not to be known to nest (53.7 pg/mL vs. 28.7 pg/mL; *F*_1,104_ = 7.860, *P* = 0.0060).

Progesterone concentrations varied both by population (*F*_2,25_ = 8.331, *P* = 0.0017) and month (*F*_11,153_ = 1.948, *P* = 0.0374). We found P averaged significantly lowest in Madagascar captives (0.44 ± 0.25 ng/mL), whereas wild and US captive females averaged similarly (1.84 ± 0.38 ng/mL and 1.44 ± 0.17 ng/mL, respectively). As seen in the E2 trends, P peaked twice (November and January) in both Madagascar populations, but again the degrees to which they occurred differed (Fig 6). Again, no distinguishable peaks could be detected in the U.S. population. Within the two captive populations, those females which were known to have laid nests had higher average P concentrations overall throughout sampling (1.05 ng/mL vs 0.57 ng/mL; *F*_1,104_ = 9.756, *P* = 0.0023).

### Body condition

**Fig 7:**
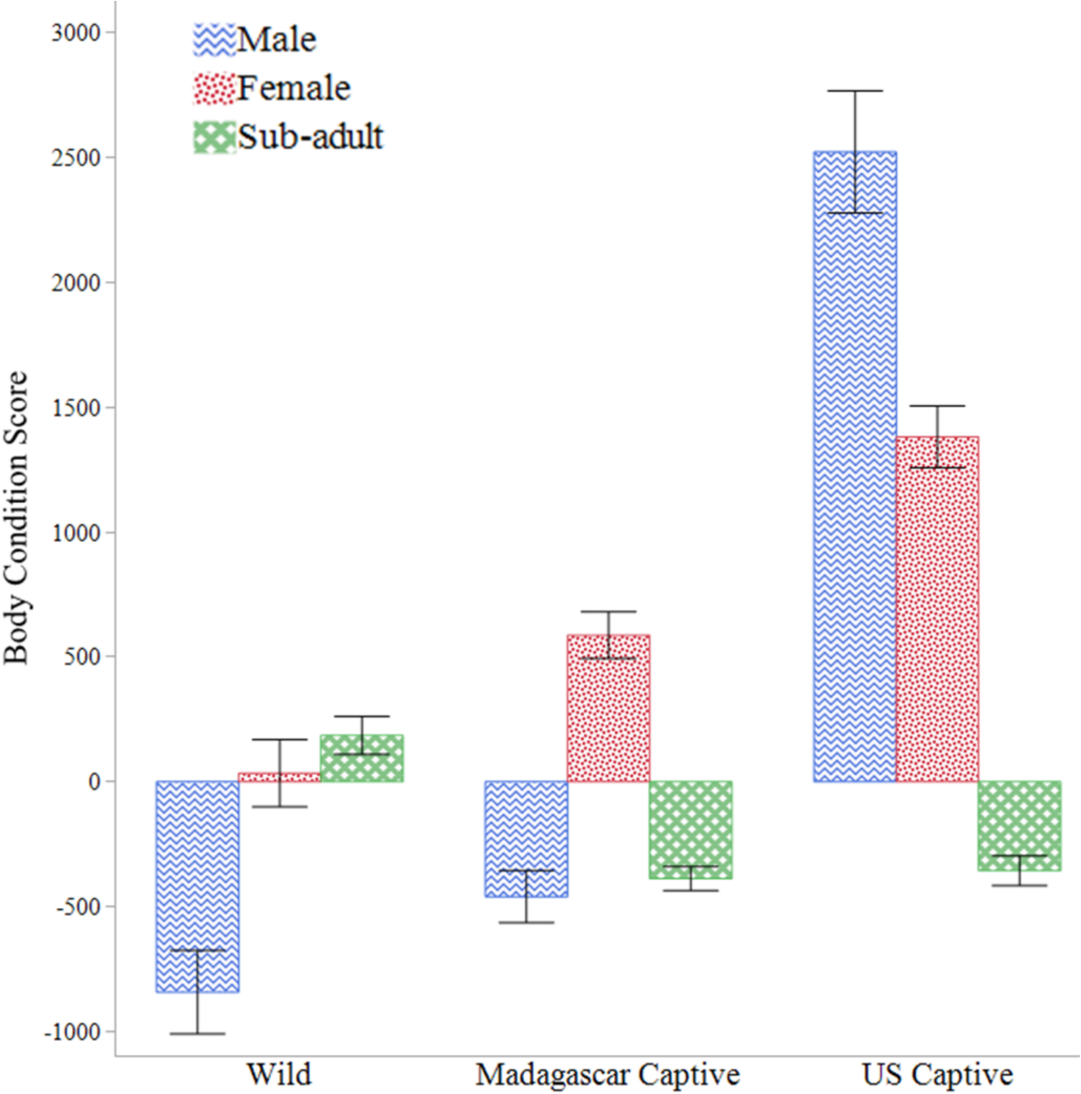
Body condition scores for *Astrochelys yniphora* populations. Body Condition Scores (residuals of weight by maximum carapace length) for each sex of *A*. *yniphora* from the wild in Madagascar, a captive population near the wild range, and a U.S. captive population from 2013–2015.

**Fig 8:**
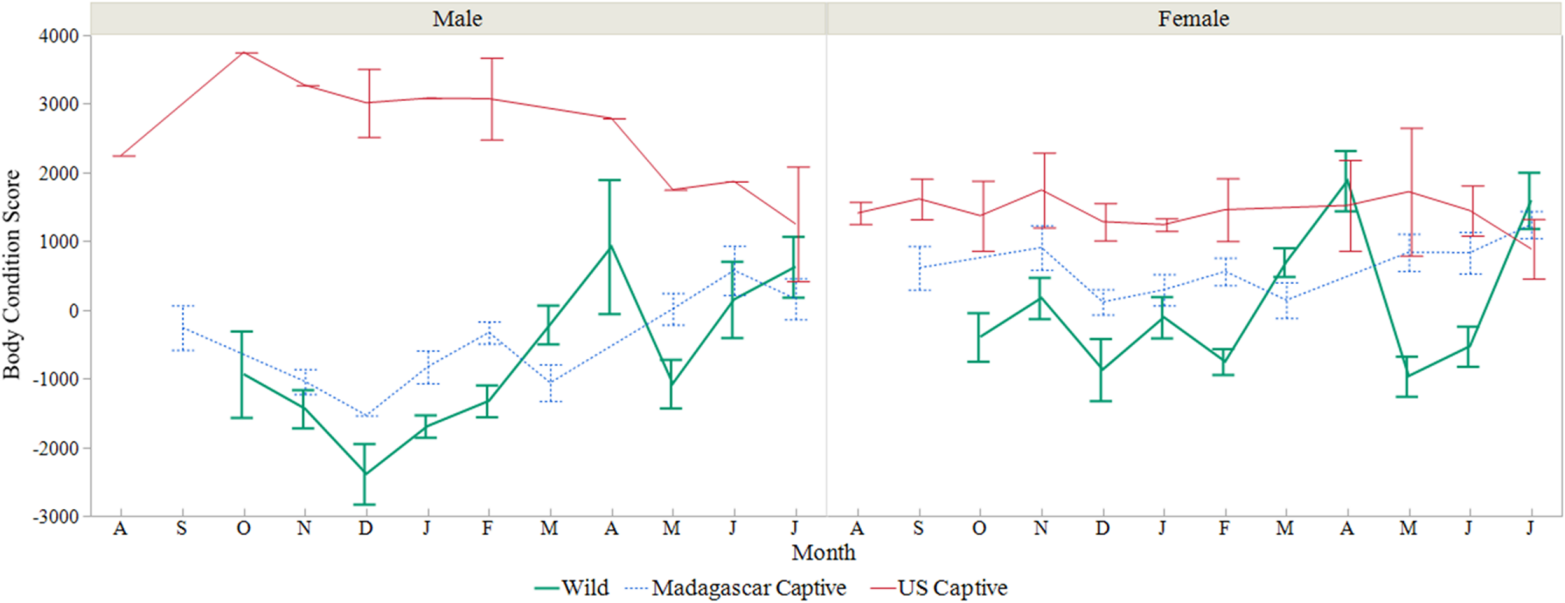
Monthly body condition scores for *Astrochelys yniphora* populations. Mean monthly body condition score (BCS; weight/length residuals) changes by month of adult male and female *A*. *yniphora* from three populations (wild, Madagascar captive, and U.S. captive). Error bars represent 1 SE.

The BCSs we calculated are summarized in Table 1. *Astrochelys yniphora* body condition varied between populations (*F*_2,127_ = 9.089, *P* = 0.0002), by sex (*F*_2,113_ = 5.832, *P* = 0.0039), and combined for populations and sexes (*F*_4,136_ = 7.640, *P* < 0.0001). Overall, females had highest scores, and significantly higher than sub-adults. Yet, the starkest difference was between the negative BCS of males from the wild populations and the highly positive BCS of the U.S. captive male (Fig 7). Adult animals in the U.S. population exhibited higher BCS than either the Madagascar populations and consistently remained highest over time (Fig 8). We did not investigate sub-adults BCSs over months between the populations due to limited samples sizes.

**Table 1:** Hormone, morphometric, and environmental data relating to *Astrochelys yniphora*.

		Wild			Madagascar Captive			U.S. Captive		
		♂	♀	SA	♂	♀	SA	♂	♀	SA
**CORT**	Mean	83.1	156.2	440.1	155.7	79.6	155.7	17.1	366.7	42.1
**(ng/dL)**	Median	28.3	47.8	135.9	47.9	29.9	47.9	11.9	157.9	6.0
	Range	3.4–1587.7	4.4–3164.1	21.1–2697.1	3.2–1597.7	1.0–1152.9	2.6–2438.1	4.1–46.7	38.4–1902.3	1.0–500.0
**Testosterone**	Mean	635.6	–	13.9	277.7	10.4	2.4	797.4	19.9	0.9
**(ng/dL)**	Median	543.3	–	2.3	41.4	10.4	0.0	1068.1	19.9	0.0
	Range	0.0–1200+	–	0.0–39.0	0.0–1200+	0.0–20.8	0–23.6	84.8–1200+	3.8–36.0	0.0–3.2
**Estradiol**	Mean	–	62.8	–	0.0	35.4	0.0	0.0	73.8	0.0
**(pg/mL)**	Median	–	45.7	–	0.0	24.9	0.0	0.0	66.2	0.0
	Range	–	0.0–198.3	–	0.0	0.0–185.1	0.0	0.0	0.0–169.0	0.0
**Progesterone**	Mean	–	1.83	–	1.15	0.68	–	–	1.50	–
**(ng/mL)**	Median	–	1.10	–	1.15	0.61	–	–	1.26	–
	Range	–	0.00–14.63	–	-	0.00–2.42	–	–	0.00–5.59	–
**Weight**	Mean	12.3	9.8	11.2	12.3	9.7	3.8	16.5	11.2	2.3
**(kg)**	Median	12.0	10.0	8.6	12.2	9.8	3.7	16.3	11.2	2.2
	Range	6.8–19.0	5.6–15.5	0.3–7.0	6.6–17.2	6.2–14.4	1.1–7.6	15.6–17.6	10.4–12.4	0.9–3.4
**MCL**	Mean	441	381	131	431	370	262	451	384	209
**(mm)**	Median	440	388	126	424	372	264	450	385	207
	Range	376–519	332–415	48–325	345–489	307–416	166–325	447–470	377–402	159–253
**Air Temp**	Mean	31.4	31.9	32.3	29.2	29.2	31.9	29.0	29.6	30.6
**(°C)**	Median	31.6	32.1	32.7	28.5	29.1	31.4	27.8	29.6	31.8
	Range	22.3–36.7	25.3–38.2	29–36.1	26.7–36.0	26.4–35.2	26.6–35.6	24.3–37.2	24.3–37.2	24.3–36.6
**Ground Temp**	Mean	32.5	33.3	34.7	29.0	29.1	31.6	29.2	29.2	34.4
**(°C)**	Median	33.3	33.3	34.3	28.8	28.3	30.9	27.6	28.5	34.7
	Range	24.2–42.5	27.5–39.0	29.6–40.0	24.6–34.7	21.7–32.6	26.9–35.4	24.8–35.1	23.7–35.1	23.7–44.0
**Ground**	Mean	68.1	67.9	66.6	71.1	71.4	68.0	50.2	48.7	48.9
**Humid (%)**	Median	67.9	65.9	71.0	73.7	66.6	67	49.0	47.9	47.9
	Range	41.4–100	35.4–100	30.2–88.7	49.5–89.9	49.5–94.6	53.2–94.6	38.3–62.5	27.3–62.5	38.3–62.5
**Cloud Cover**	Mean	35.7	35.8	26.0	46.6	45.5	34.6	10.0	7.0	15.0
**(%)**	Median	20.0	30.0	10.0	50.0	50.0	0.0	0.0	0.0	15.0
	Range	0–100	0–100	0–100	0–100	0–100	0–100	0–40	0–40	0–30
**Veg Cover**	Mean	68.7	64.0	58.0	38.8	47.7	68.8	46.6	47.5	33.8
**(%)**	Median	70.0	60.0	65.0	30.0	40.0	80.0	47.5	50.0	30.0
	Range	10–100	10–100	10–100	10–100	10–80	20–90	40–60	30–60	30–60

Mean, median, and range (min–max) circulating hormone concentrations, weights, maximum carapace length (MCL), air and ground temperatures, ground humidity, and cloud and vegetation cover relating to male (♂), female (♀), and sub-adult (SA) *Astrochelys yniphora* from the wild, a captive population near native range in Madagascar, and a captive population in the U.S. collected from 2013–2015.

For females within the native country, BCSs were generally highest heading into the dry season (July), recovering after a drop, presumably due to nesting. Malagasy males, too, increased into July after an evident dip into the onset of the wet season (October–December).

### Environment and behaviors

**Fig 9:**
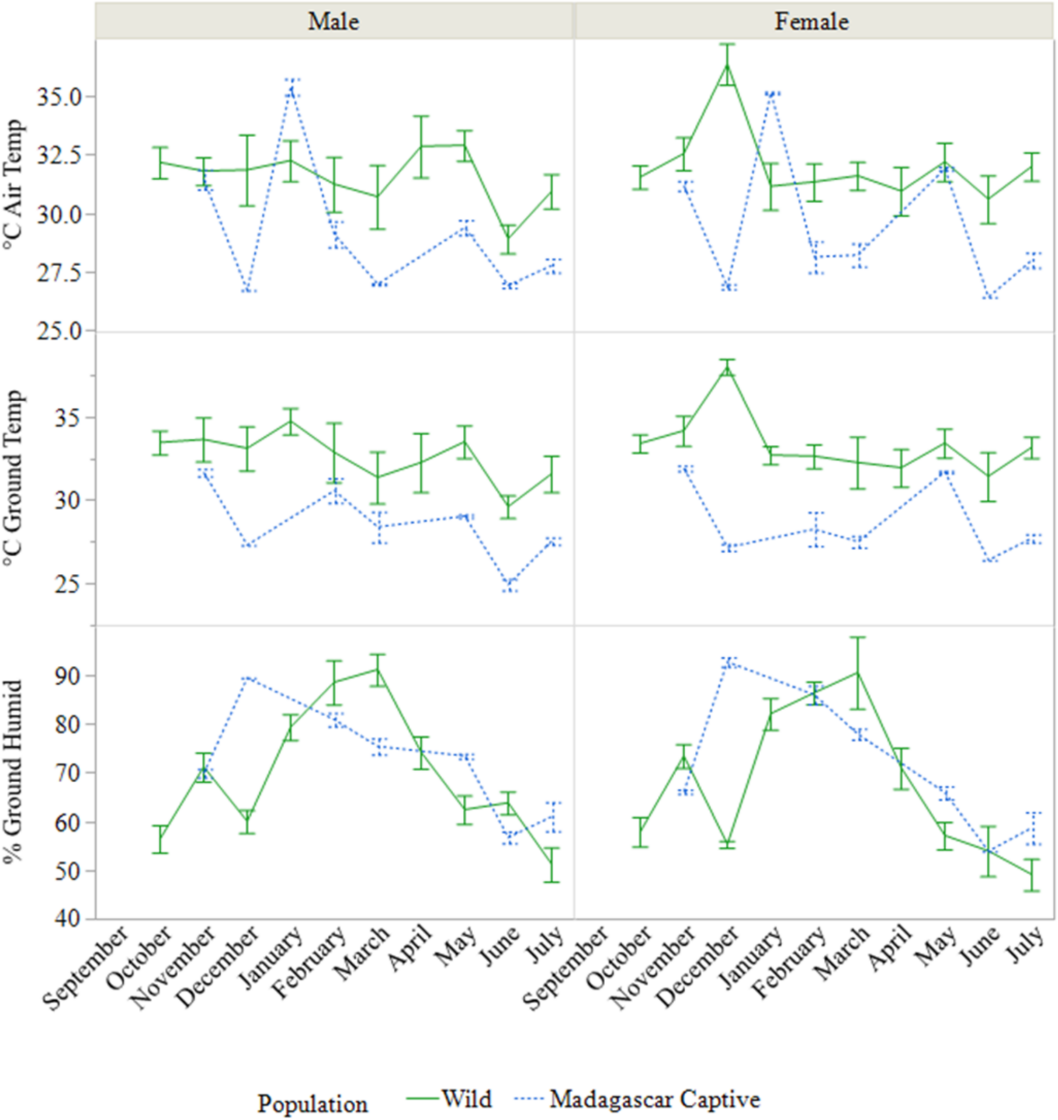
Environmental factors associated with *Astrochelys yniphora*. Mean air temperature, ground temperature, and ground humidity by month and sex at *A*. *yniphora* locations between the two Madagascar populations from 2012–2015.

Air temperatures, ground temperatures, humidities, and cloud cover were significantly different between months and populations (all *P* < 0.0001; Table 1). Temperatures selected for by animals (ground temperatures) in the wild population remained relatively constant over time (averaging 33.2°C) with a couple exceptions that correspond to other measurable changes in hormones and BCS, and were generally higher overall than the captive populations. The selected monthly temperatures varied more widely in both captive populations than the wild population (Fig 9). In all populations, males preferred overall cooler temperatures than females or sub-adults, with the highest temperatures preferred by sub-adults (Table 1). Vegetation cover where animals were found was different between months (e.g., seasonality; *F*_11,254_ = 3.974, *P* < 0.0001), but not between populations.

**Fig 10:**
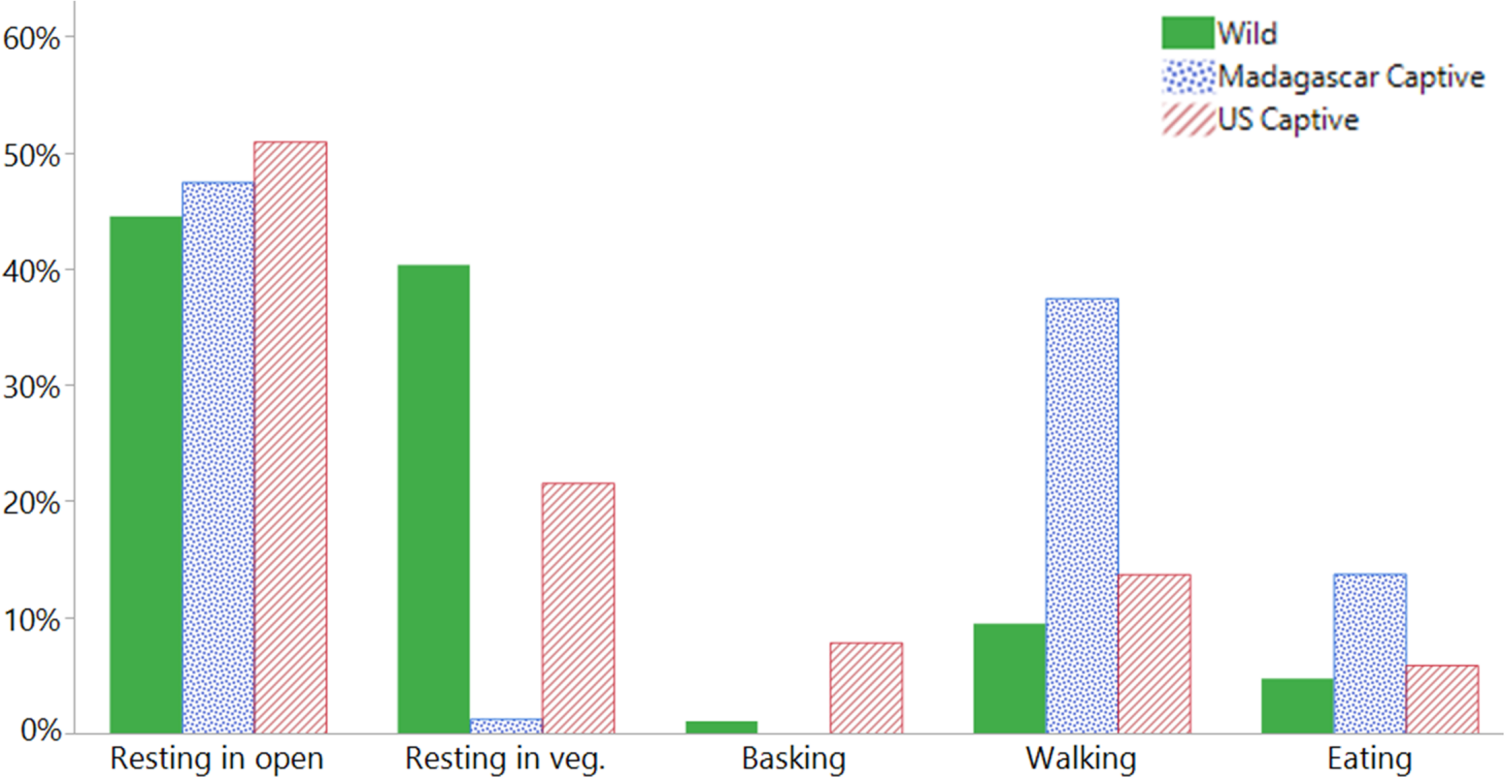
Observed activity of *Astrochelys yniphora* populations. Percent of total observed activity of *A*. *yniphora* from three populations (wild, Madagascar Captive, and U.S. Captive).

We detected differences in the distribution of activity exhibited by the animals between the populations (L-R *χ*^2^ = 135.606, df = 10, *P* < 0.0001; Fig 10), but not between the sexes. Concordantly, observed animal activity also differed by ground humidity (*χ*^2^ = 17.631, df = 4, *P* = 0.0015), cloud cover (*χ*^2^ = 11.840, df = 4, *P* = 0.0186), and vegetation cover (*χ*^2^ = 15.747, df = 4, *P* = 0.0034). When separated by population, we further found differences. Animals in the wild were more often found to be under vegetation than either of the captive populations, while the Madagascar captive were more often eating and walking (Fig 10). Of the variables measured, wild *A*. *yniphora* activity was most affected by ground temperature and humidity (*χ*^2^ = 62.062, df = 32, *P* = 0.0011); where low temperatures (32.8 ± 0.37°C) and humidities (62.1 ± 1.6%) were associated with resting in vegetation while higher temperatures (33.7 ± 0.8°C) were associated with eating and resting in the open, and higher humidities (74.3 ± 2.7%) were associated with resting in the open and walking. For the Madagascar captives, we found that ground humidity, cloud cover, and vegetation cover had significant effects on activity (*χ*^2^ = 40.553, df = 21, *P* = 0.0064). Madagascar captives would more often be walking in higher humidity (75.0 ± 1.9%) and vegetation cover (56.7 ± 3.7%), resting in the open when cloud cover was higher (53.7 ± 3.2%), and eating and resting in the vegetation in lower cloud cover (33.8 ± 13.8%) and vegetation covered areas (35.8 ± 11.4%). In the U.S. captive population, *A*. *yniphora* activity was most affected by air temperature (*χ*^2^ = 8318, df = 2, *P* = 0.0156). U.S. captive animals were more often resting in the open or walking in higher temperatures (32.2 ± 2.0°C), and eating, basking, or exhibiting reproductive behaviors in cooler temperatures (25.2 ± 2.5°C).

We investigated environmental variables’ influence on activity by month in the Madagascar populations for which we had enough data. In the month of December, wild *A*. *yniphora* behaviors depended on ground temperatures, finding warmer temperatures (37.5 ± 1.0°C) in vegetation as opposed to in the open (32.5 ± 1.0°C; *F*_3,12_ = 3.826, *P* = 0.0391). They were found eating and walking much more in February when there was high cloud cover (70–90 ± 20.6%) opposed to only resting when cloud cover was low (22–24 ± 9.0%; *F*_3,15_ = 4.300, *P* = 0.0223). For the Madagascar captive population, air and ground temperatures and ground humidity in November separated walking (35.5 ± 0.5°C, 64.1 ± 1.2%) and resting activities (32.5 ± 0.3°C, 53.2 ± 1.8%; all *F*_1,21_ = 20.0–25.8, *P* ≤ 0.0002). In May, the Madagascar captives’ activity was also associated with environment, where air temperature, ground temperature, cloud cover, and vegetation cover, resting in higher temperatures and vegetation cover under lower cloud cover (all *P* ≤ 0.0172).

## Discussion

### Wild vs captives: Ecophysiological and reproductive trends

Captive animals have been reported to be heavier (kg) than their wild counterparts [73]. In the present study, animals in the wild population exhibited BCSs nearest to zero, while both captive populations differed more widely. Captive adults generally had more positive BCSs than those in the wild; however, sub-adults showed the opposite trend. In captivity, animals are usually allowed free access to perpetual food and water resources, and adults are housed in less crowded enclosures, separated from sub-adults. It is possible that interspecific competition and boldness/shyness of individuals among the sub-adults in more crowded enclosures account for the lower BCSs and consistent stress within that group (e.g., [30, 74]). In a study on great tits (*Parus major*), animals which exhibited shyness behaviors were also those same animals which exhibited higher basal CORT concentrations along with more extreme stress responses [75]. A study on curve-billed thrashers (*Toxostoma curvirostre*) showed that animals exhibited an increase in basal CORT values when food-restricted in captivity [76]. In the present study, an additional factor should be considered: sub-adults in the Madagascar captive population are generally fed native forage species; however, there are no studies on what sub-adults consume in the wild and how it may differ from adults and by season. In many chelonian species, sub-adults and adults not only inhabit differing habitats, but their diets are composed of different ratios or of entirely different species/items [77–81]. Therefore, the BCS differences we see could involve factors for which we have not investigated here, and further research should be done.

Though high CORT is often seen in wild-caught animals in captivity, captive-bred animals generally do better, exhibiting little to no chronic stress or attenuated stress responses [7, 82, 83]. Consistent with those studies, we found that the highest CORT concentrations were exhibited by wild sub-adults. In the wild, elevated CORT may be adaptive because it is associated with predator avoidance behaviors and cautious animal personality types in other species [21, 74, 84], including the most closely related sister taxa to *A*. *yniphora*, *A*. *radiata* [85]. Although variability might still be harbored in the populations, captive sub-adults would not be exposed to such stressors and would be less likely to undergo such a response. These naiveties can be detrimental to survival [86]. In a study on multiple parrot species, researchers found that most captive-bred birds, but only a small percentage of wild birds, were either recaptured or preyed on shortly after escape from enclosures [82].

Elevated CORT is associated with predator avoidance behaviors and cautious animal personality types, and sub-adults in captivity generally do not exhibit a strong stress response or elevated basal CORT (as we found in the current study). In fact, individuals exhibiting aggressive behaviors or which are otherwise obviously stressed by captivity are generally bred out or avoided in captive breeding programs. This may be detrimental to individual survival because when the captive-bred animals are released into the wild they are unable to recognize threats and respond appropriately [1, 86, 87]. As a supporting anecdote, we visited captive-bred *A*. *yniphora* that were repatriated to a previously depleted site within the natural range [88, 89]. When those animals are encountered in the wild, they readily approach humans, do not turn away or attempt to retreat when handled, and extend their limbs and neck if stroked or scratched (ATFC and ARM, pers. obs.). Though bold individuals may be avoided or bred out in captivity, the genetic variation they represent may be important for the long-term survival of a species which needs to adapt to changing habitats, threats, and climate. Variability provides the population an opportunity to remain reproductively viable amid stochastic events and face novel situations. Maintaining variability will also help avoid inbreeding depression in other ways, such as in preserving healthy immune system functions. Understanding and mediating stressors are therefore an important part of conservation management.

Captivity has been shown to elicit a chronic stress response in many wild-caught animal species [75, 76, 90, 91]; but see [92]). Therefore, as might be expected, adult CORT concentrations were generally higher in captive populations than the wild, yet were not consistent. Only the captive males housed within the native country were higher, while the single male in the U.S. was lowest. The opposite trend was found in females, but were within range of the wild animals. The distinct values found in the U.S. population were probably due to specific circumstances associated with those individuals and are described by sex separately below.

#### Males

The U.S. population’s adult male scored the highest positive BCSs, but males from both the Madagascar populations showed mean negative BCSs. Over time, both the Madagascar populations’ males exhibited similar BCS trends to one another, peaking mid-dry-season in July, which is likely associated with growth of testicular tissue (spermiogenesis and/or spermiation) as a surge in T occurs at that time. In many herpetofauna, reproductive timing in males is altogether or partially offset from mating season [93, 94], and this appears to be true in *A*. *yniphora* as well. Courtship and mating are seasonal in ploughshares, and generally occurs from October through December at the onset of the region’s wet season. Body condition scores dipped to the lowest values during the early wet season, when male-male competition for mates would be peaking. It has been suggested that males need to engage in combat in order to trigger their sexual interest in females [67], and the combat itself will cause males to take in fewer food and water resources, altering BCSs. Males housed in the Madagascar captive population are regularly placed in shared enclosures, often specifically for these combat rituals, which may also help explain why they expressed BCSs and T concentrations more similar to wild individuals. Because the Madagascar captive population is generally fed native plant material, there may be a nutritional component to the similarity, as well as environmental, behavioral, and physiological components.

In the wild population, males exhibit two CORT peaks which align with the time of ovulation in females, suggesting that competition for (and possibly defense of) mates is stressful for males. Monthly CORT in captive males was much more variable, and may be due to the changing of pairings and enclosures. However, we found that captive males which sired nests had overall higher basal CORT and T concentrations, suggesting that male-male competition is both stressful and requires aggression/dominance throughout the year. Similarly, in the eastern black rhinoceros (*Diceros bicornis michaeli*), dominant males exhibited significantly higher T and became sires by monopolizing more matings than non-sires [95]. Although male sires in the current study had higher overall mean T, they exhibited significantly lower T during the months for which they sired. It logically follows that this strategy would be adaptive. For species which competition determines mating rights, aggressive males with higher T would generally be more successful in combat, but those T-linked aggressive behaviors could attenuate successful mating if directed at mates themselves. Dominant male *A*. *yniphora* may therefore undergo a reduction in T concentrations after combat to avoid misdirecting overly aggressive behaviors (e.g., causing harm or refusal by mates) and consequently, for courtship to take place.

Nearly half of all the T samples collected from the U.S. captive male *A*. *yniphora* were extremely high for much of the year (Fig 5). The U.S. male is not housed with other adult males and without the need to compete for access to females. His CORT concentrations remain low throughout the year while his T concentrations remain high throughout courtship, and aggression toward the females was noted. Although the females to which the U.S. male has access nested multiple times during this study, no fertilized eggs have been detected and sperm were not present in two eggs which were sacrificed to inspect them for the presence of sperm [96]. This single example is concordant with the data presented above, suggesting that male *A*. *yniphora* may need to reduce T concentrations (through increasing CORT or otherwise) to successfully reproduce. Yet, there is still debate on what the variation in T signals with regard to an individual’s biology, and investigations into the relative importance and extrinsic influences that contribute to variation need be conducted to better interpret variation in T [97].

#### Females

Female ploughshares produce 2–5 eggs per clutch, and have been seen to clutch multiply in both captivity and in the wild [61]. They have been reported to produce an average of 1.5–6.4 clutches depending on individual and location, but individuals in captivity produce both more clutches and more eggs per clutch than their wild counterparts as well as extend their nesting period into later months [61]. Increases in E2 are associated with vitellogenesis and ovulation, while P peaks post-ovulation [62]. These peaks may be within days of each other, and our monthly sampling regime was not frequent enough to detect these changes in individuals. On the population-level, however, it is general practice to describe seasonal cycles as monthly trends in reproductive hormones, usually only over the months known for reproductive activity [62, 69, 93, 98]. We were able to collect data for every month of the year (though, not for every population), and we do see distinct cycle trends among population in female BCS, CORT, E2, and P on an annual basis.

Female chelonians exhibit increased weights at the time when follicular development/vitellogenesis occurs. Concordantly, mean BCSs in adult females from all three populations were found to be positive, and changes in BCS over time appear to reflect nesting events. Both captive populations exhibited higher BCSs in adult females than the wild; however, monthly trends were less evident than in wild females. It has been shown that captive female *A*. *yniphora* housed in Madagascar lay more clutches than wild *A*. *yniphora* [61] which may be due to access to mates stimulating ovarian development [60]. Therefore, the difference in captive females’ BCSs were probably not only due to restricted vagility alongside constant access to food and water resources, but probably also due to increased clutches per year. The idea that females may need to be paired with males for ovulation to occur suggests that: 1) this species may not have the capacity for sperm storage as is seen in many other chelonians and 2) the declining population density could directly affect reproductive output, exacerbating declines (as was seen in the passenger pigeon). For chelonian species where both vagility and density are low, the ability to store viable sperm from one season to the next is an incredible adaptation maintaining species survival. Unfortunately for the ploughshare, they may not have this ability yet, but it should be further investigated.

In the current study, general monthly hormone trends of female *A*. *yniphora* displayed synchronous upticks in CORT, E2, and P which were followed by decreases in BCSs. For the captive populations, these trends often lined up with observations of nesting. Generally, females exhibited two peaks in E2 and P. For wild females, E2 and P exhibited one smaller peak two months prior to a larger peak, suggesting that two clutches may be produced annually in the wild which is concordant with the study on nesting behavior by [61]. The two hormone peaks we note in females align with the two CORT peaks in wild males, and we also see the strongest uptick in female CORT along with those second peaks. Higher basal CORT concentrations have been associated with mating other species; both the male and female degu (*Octodon degus*) exhibit higher CORT in captivity and in the wild [92]. It begs the question as to whether *A*. *yniphora* are encountering or seeking out each other more often during those peak times. Further, female *A*. *yniphora* may not ovulate without having been paired with a male during the previous mating season, despite having preovulatory follicles [60].

We found that CORT was highest in females from the U.S. captive population, and consistently lowest in the Madagascar captive population. It is likely that these trends are directly related to their particular captive conditions. Females from the Madagascar captive population are separated from males in their group enclosures during all times except for matings. The two females in the U.S. population are relatively new imports to the facility and are offered little retreat from the males’ advances with which they are regularly housed. Those U.S. females’ E2 and P appear completely disrupted without clear peaks. Female reproductive cycles may become disrupted for a variety of reasons, and captivity may delay the onset on ovulation by months or years. Though some species have the capacity to attenuate reproduction in any given year, the underlying physiological regulation of this ability is not well understood [99]. It is probable that the females’ cycles are affected by the transfer into the northern hemisphere and are not yet acclimatized to the change. Though there are possible acclimation and persistent courtship stressors, the females are well fed and undergoing periods of follicular development [100] and nesting events. We further discuss the acclimation issues in the Environmental Considerations section below.

### Age-class challenges

One of the challenges of captive breeding long-lived, slowly maturing species with ambiguous phenotypic sex differences is planning effective releases which will offer optimal sex ratios. Ploughshares reach sexual maturity at approximately 300 mm carapace length, and males can be distinguished from females using secondary sexual characteristics at about 350 mm carapace length [48]. Size and age are generally correlated in chelonians, and those animals from 300–350 mm carapace length are estimated to be approximately 20–25 years old. If the sex of an animal is unknown for 15–25 years, as in this and many other chelonian species, a conservation strategy may be severely impaired or carried out without enough information to be successful. This has been an ongoing challenge for chelonian conservation management plans in general [101, 102]. One option for premature sexing of chelonians is the investigation of circulating hormone concentrations. It has been suggested that T concentrations may be used a biomarker to distinguish between the sexes in sub-adult chelonians and has been used successfully in *Caretta caretta* (loggerhead sea turtles) and *Gopherus agassizii* (desert tortoises; [103–105]).

In the present study, wild sub-adults could be distinguished from wild males via higher CORT and lower T. The trend of lower T than adult males is carried over among all the study populations. Plasma T concentrations were no different in sub-adults than females, but were significantly different than adult males. This suggests that sub-adult individuals exhibiting high T concentrations, especially during peak T months for adult males, could be assigned as male. In this species, it appears as though any T detection higher than 20 ng/mL from a sub-adult during the months October–November housed in Madagascar, or one housed in the U.S. during April–May, could be assigned as male relatively confidently; however, we caution that more directed investigations be done to hone the method. Additionally, if female hormones are to be investigated for this purpose, we suggest using E2 over P as the single male sample we ran for both hormones did exhibit detectable P, but not E2.

### Environmental considerations

There were significant behavioral associations with every environmental variable we measured. Our findings suggest that animals seek out different conditions for varying activities and do so at differing times of the year. Most consistently, however, temperature, humidity, and cover appear to be the most important variables.

Temperature appears to regulate the production on estrogens in chelonians [78, 106, 107]. In the present study, we found that wild females were able to find significantly warmer locations resting in vegetation in December than males (which were usually found resting in the open), and that correlated with a dip in CORT, E2, and P. Less difference was seen in the humidities females selected, and overall, the wild animals selected warmer temperatures than the captives. Parturition has been documented in this species both in the wild at one of our sample sites and in our Madagascar captive population from 1995–2000 [61]. According to [61] found *A*. *yniphora* nested multiply, averaging 2.1 clutches per animal per year in the wild and 5.4 clutches per animal per year in captivity. Although our data from the wild population support those findings, the timing of nesting over the 15 years since that study appears to have shifted to earlier in the year, and during a time of year (November) when data is lacking completely in other studies [60, 61, 108]. If reproduction in this species is indeed tied to environmental cues, this apparent shift in reproductive timing of this species could be attributed to climate change.

Environmental variables should be carefully considered when ectotherms are to be moved for assurance colonies and breeding. The hemispheric differences in climate we note between the U.S. and Madagascar captives has had a dramatic effect on the animals, and those that are housed in the U.S. are still not yet in successful reproductive condition. Though similar to the Madagascar populations where a seasonal dip and peak in BCS can be noted in the male housed in the U.S., the timing of the changes were temporally shifted by about five months forward (e.g., July instead of December). The U.S.-housed male’s reproductive hormones are most extreme and opposite of those in the Madagascar populations, while the U.S. females’ appear to be completely disrupted. Each of the adult animals in that population was transferred to the U.S. at a different month and year. The lack of reproductive success may be that the animals’ internal reproductive timing has not yet adjusted to the climate of southern California, which is in opposing seasonality with Madagascar and southern hemisphere. Those individuals housed in captivity in Madagascar are near their native range and did not undergo a significant delay before successful reproduction began. Using the hormone profiles presented in the current paper, it appears that the U.S. captives may be undergoing shifts in reproductive timing concordant with environmental cues. It is apparent that seasonal reproductive cycles are not easily or instantly acclimatized to large seasonal shifts. It may be several years before those translocated animals synchronize their reproductive cycles.

## Conclusions

Wild animal studies often feature a suite of challenges and complexity which may confound or limit the scope of interpretation. Yet, it is of great importance that longer-term, comparative studies take place and be published so that physiological details and correlated ecological consequences can be further understood, improving both *in-situ* and *ex-situ* species conservation management. This study was conducted at an extremely opportune time for this particular CR ectotherm as it was completed just prior to probable extirpation in the wild. Though we do not include complete captive husbandry protocols, the data presented here are intended for immediate application for improving captive conditions and species survival. We aimed to collect as much ecological, physiological, and behavioral data as possible from wild populations of a CR ectotherm to create a detailed picture of natural conditions, requirements, and baselines. With these combined data, we increase our power of inference, but also expose wide variation and plasticity. A limited view into this variation may narrow conclusions about the reproductive capacity of a population or the species. Individuals that are merely surviving but not thriving, (as appears to be the case in the U.S. captive population) will be the failure of breeding programs, reintroductions, and possibly species survival if not identified and rectified. Our results indicate that long-lived ectothermic species require certain social, behavioral, and environmental cues for successful reproduction. We show that detailed ecophysiological data should be used when creating and improving captive husbandry conditions for conservation breeding programs.

## Supporting information

S1 Table Raw dataset. All data underlying the findings described in this manuscript. These are the data points behind means, medians, and variance measures presented in the manuscript by Currylow et al., “*Comparative ecophysiology of a Critically Endangered (CR) ectotherm*: *implications for conservation management*”.
(PDF)

## References

[1] Noel FRS, Derrickson SR, Beissinger SR, Wiley JW, Smith TB, Toone WD, et al Limitations of captive breeding in endangered species recovery. Conserv Biol. 1996;10(2):338–48.

[2] Dodd CK Jr., Seigel RA. Relocation, Repatriation, and Translocation of Amphibians and Reptiles—Are They Conservation Strategies That Work. Herpetologica. 1991;47(3):336–50.

[3] Seal U, Foose T, Ellis S. Conservation assessment and management plans (CAMPs) and global captive action plans (GCAPs) Creative Conservation: Springer; 1994 p. 312–25.

[4] Magin C, Johnson T, Groombridge B, Jenkins M, Smith H. Species extinctions, endangerment and captive breeding Creative conservation: Springer; 1994 p. 3–31.

[5] Millam JR, Roudybush TE, Grau CR. Influence of environmental manipulation and nest-box availability on reproductive success of captive cockatiels (Nymphicus hollandicus). Zoo Biol. 1988;7(1):25–34.

[6] Shepherdson DJ, Mellen JD, Hutchins M. Second nature: Environmental enrichment for captive animals: Smithsonian Institution; 2012.

[7] Mason G, Burn CC, Dallaire JA, Kroshko J, McDonald Kinkaid H, Jeschke JM. Plastic animals in cages: behavioural flexibility and responses to captivity. Anim Behav. 2013;85(5):1113–26.

[8] Greenberg N, Wingfield J. Stress and reproduction: reciprocal relationships. Hormones and reproduction in fishes, amphibians and reptiles Plenum, New York 1987 p. 461–503.

[9] Guillette LJ Jr., Cree A, Rooney AA. Biology of stress: interactions with reproduction, immunology and intermediary metabolism. Health and Welfare of Captive Reptiles. 1995:32–81.

[10] Wolf KN, Wildt DE, Vargas A, Marinari PE, Ottinger MA, Howard JG. Reproductive inefficiency in male black-footed ferrets (Mustela nigripes). Zoo Biol. 2000;19(6):517–28. doi: 10.1002/1098-2361(2000)19:6<517::AID-ZOO4>3.0.CO;2-V 11180413 10.1002/1098-2361(2000)19:6<517::AID-ZOO4>3.0.CO;2-V

[11] Dobson A, Lyles A. Black-Footed Ferret Recovery. Science. 2000;288(5468):985–8. 10841720 10.1126/science.288.5468.985

[12] Seal US, Thorne ET. Conservation biology and the black-footed ferret. New Haven, Connecticut, USA: Yale University Press; 1989.

[13] Snyder NF, Snyder H. The California Condor: a saga of natural history and conservation. San Diego, California, USA: Academic Press; 2000. 410 p.

[14] Heppell SS, Crowder LB, Crouse DT. Models to evaluate headstarting as a management tool for long-lived turtles. Ecol Appl. 1996;6(2):556–65.

[15] Henen BT. Seasonal and annual energy budgets of female Desert Tortoises (*Gopherus agassizii*). Ecology. 1997;78(1):283–96.

[16] Amo L, López P, Martín J. Habitat deterioration affects body condition of lizards: a behavioral approach with *Iberolacerta cyreni* lizards inhabiting ski resorts. Biol Conserv. 2007;135(1):77–85.

[17] Ewenson EL, Zann RA, Flannery GR. Body condition and immune response in wild Zebra Finches: effects of capture, confinement and captive-rearing. Naturwissenschaften. 2001;88(9):391–4. 11688415 10.1007/s001140100250

[18] Sapolsky RM. Neuroendocrinology of the stress response In: Becker JB, Breedlove SM, Crews D, editors. Behavioral Endocrinology. Cambridge, MA and London: MIT Press; 1992 p. 287–324.

[19] Wingfield JC, Donna LM, Creagh WB, Jacobs JD, Sharon L, Ramenofsky M, et al Ecological bases of hormone-behavior interactions: the "emergency life history stage". Am Zool. 1998;38(1):191–206.

[20] Sapolsky RM, Romero LM, Munck AU. How Do Glucocorticoids Influence Stress Responses? Integrating Permissive, Suppressive, Stimulatory, and Preparative Actions. Endocr Rev. 2000;21(1):55–89. doi: 10.1210/edrv.21.1.0389 10696570 10.1210/edrv.21.1.0389

[21] Moore IT, Jessop TS. Stress, reproduction, and adrenocortical modulation in amphibians and reptiles. Horm Behav. 2003;43(1):39–47. 12614633 10.1016/s0018-506x(02)00038-7

[22] Mahmoud I, Guillette L Jr, McAsey M, Cady C. Stress-induced changes in serum testosterone, estradiol-17*β* and progesterone in the turtle, *Chelydra serpentina*. Comparative Biochemistry and Physiology Part A: Physiology. 1989;93(2):423–7.

[23] Blas J, Bortolotti GR, Tella JL, Baos R, Marchant TA. Stress response during development predicts fitness in a wild, long lived vertebrate. Proceedings of the National Academy of Sciences. 2007;104(21):8880. 10.1073/pnas.0700232104 PMC1868653 17517658

[24] Gregory LF, Schmid JR. Stress responses and sexing of wild Kemp's Ridley Sea Turtles (*Lepidochelys kempii*) in the northeastern Gulf of Mexico. Gen Comp Endocrinol. 2001;124(1):66–74. doi: 10.1006/gcen.2001.7683 11703072 10.1006/gcen.2001.7683

[25] Abbott DH, Keverne EB, Bercovitch FB, Shively CA, Mendoza SP, Saltzman W, et al Are subordinates always stressed? A comparative analysis of rank differences in cortisol levels among primates. Horm Behav. 2003;43(1):67–82. 12614636 10.1016/s0018-506x(02)00037-5

[26] Norris DO, Lopez KH, editors. Hormones and Reproduction of Vertebrates. 1st ed. San Diego, CA, USA: Academic Press, Elsevier; 2011.

[27] Balm PH. Stress Physiology in Animals. Sheffield, England: Sheffield Academic Press Ltd.; 1999.

[28] Romero LM. Physiological stress in ecology: lessons from biomedical research. Trends Ecol Evol. 2004;19(5):249–55. doi: 10.1016/j.tree.2004.03.008 16701264 10.1016/j.tree.2004.03.008

[29] Romero LM, Reed JM. Collecting baseline corticosterone samples in the field: is under 3 min good enough? Comparative Biochemistry and Physiology-Part A: Molecular & Integrative Physiology. 2005;140(1):73–9. 10.1016/j.cbpb.2004.11.004 15664315

[30] Morgan KN, Tromborg CT. Sources of stress in captivity. Appl Anim Behav Sci. 2007;102(3–4):262–302.

[31] Broom DM. Animal welfare: concepts and measurement. J Anim Sci. 1991;69(10):4167–75. 1778832 10.2527/1991.69104167x

[32] Weary DM, Huzzey JM, von Keyserlingk MAG. Board-invited review: using behavior to predict and identify ill health in animals. J Anim Sci. 2009;87(2):770–7. doi: 10.2527/jas.2008-1297 18952731 10.2527/jas.2008-1297

[33] Ullman-Culleré MH, Foltz CJ. Body condition scoring: a rapid and accurate method for assessing health status in mice. Comparative Medicine. 1999;49(3):319–23. 10403450

[34] Currylow AF, MacGowan BJ, Williams RN. Short-term forest management effects on a long-lived ectotherm. PLoS ONE. 2012;7(7):e40473 doi: 10.1371/journal.pone.0040473 22792344 10.1371/journal.pone.0040473 PMC3391286

[35] Kern P, Cramp RL, Franklin CE. Physiological responses of ectotherms to daily temperature variation. J Exp Biol. 2015;218(Pt 19):3068–76. doi: 10.1242/jeb.123166 26254318 10.1242/jeb.123166

[36] Dupoué A, Brischoux F, Lourdais O, Angelier F. Influence of temperature on the corticosterone stress-response: an experiment in the Children's python (*Antaresia childreni*). Gen Comp Endocrinol. 2013;193:178–84. doi: 10.1016/j.ygcen.2013.08.004 23948369 10.1016/j.ygcen.2013.08.004

[37] Stevenson RD, Peterson CR, Tsuji JS. Thermal dependence of locomotion, tongue flicking, digestion, and oxygen consumption in the Wandering Garter Snake. Physiol Zool. 1985;58:46–57.

[38] Gans C, Crews D, editors. Hormones, Brain, and Behavior: University of Chicago Press; 1992.

[39] Spinks PQ, Pauly GB, Crayon JJ, Shaffer HB. Survival of the western pond turtle (*Emys marmorata*) in an urban California environment. Biol Conserv. 2003;113:257–67.

[40] Gibbs JP, Hunter EA, Shoemaker KT, Tapia WH, Cayot LJ. Demographic outcomes and ecosystem implications of giant tortoise reintroduction to Espanola Island, Galapagos. PLoS ONE. 2014;9(10):e110742 doi: 10.1371/journal.pone.0110742 25350744 10.1371/journal.pone.0110742 PMC4211691

[41] Santana FE, Swaisgood RR, Lemm JM, Fisher RN, Clark RW. Chilled frogs are hot: hibernation and reproduction of the Endangered Mountain Yellow-legged Frog *Rana muscosa*. Endanger Spec Res. 2015;27(1):43–51.

[42] Griffiths RA, Pavajeau L. Captive breeding, reintroduction, and the conservation of amphibians. Conserv Biol. 2008;22(4):852–61. doi: 10.1111/j.1523-1739.2008.00967.x 18616746 10.1111/j.1523-1739.2008.00967.x

[43] Rosier RL, Langkilde T. Does environmental enrichment really matter? A case study using the Eastern Fence Lizard, *Sceloporus undulatus*. Appl Anim Behav Sci. 2011;131(1–2):71–6.

[44] Jenkins RKB, Tognelli MF, Bowles P, Cox N, Brown JL, Chan L, et al Extinction risks and the conservation of Madagascar's reptiles. PLoS ONE. 2014;9(8):e100173 doi: 10.1371/journal.pone.0100173 25111137 10.1371/journal.pone.0100173 PMC4128600

[45] Walker R, Lewis R, Mandimbihasina A, Goode E, Gibbons P, Currylow A, et al The conservation of the world’s most threatened tortoise: the Ploughshare Tortoise (*Astrochelys yniphora*) of Madagascar. Testudo. 2015:68–75.

[46] Kiester AR, Mandimbihasina A, Lewis R, Goode E, Juvik J, Young R, et al Conservation of the Angonoka (Ploughshare Tortoise), *Astrochelys yniphora*. Chelonian Res Monogr. 2013;6:162–70.

[47] Curl DA. The rarest tortoise on earth. Oryx. 1986;20(1):35–9.

[48] Mandimbihasina A, Currylow A. New data on the naturally-occurring maximum sizes attained by Ploughshare Tortoises (*Astrochelys yniphora*). Herpetology Notes. 2014;7:685–8.

[49] Leuteritz TEJ, Pedrono M. Astrochelys yniphora. In: Castellano CM, Rhodin AG, Ogle M, Mittermeier RA, Randriamahazo H, Hudson R, et al., editors. Turtles on the Brink in Madagascar: Proceedings of Two Workshops on the Status, Conservation, and Biology of Malagasy Tortoises and Freshwater Turtles: Chelonian Research Foundation; 2013.

[50] Smith LL, Reid D, Robert B, Joby M, Clément S. Status and distribution of the angonoka tortoise (*Geochelone yniphora*) of western Madagascar. Biol Conserv. 1999;91(1):23–33.

[51] Juvik J, Andrianarivo A, Blanc C. The ecology and status of Geochelone yniphora: a critically endangered tortoise in northwestern Madagascar. Biol Conserv. 1981;19(4):297–316.

[52] Juvik JO, Kiester AR, Reid D, Coblentz B, Hoffman J, editors. The conservation biology of the angonoka, Geochelone yniphora, in northwestern Madagascar: Progress Report. Conservation, Restoration, and Management of Tortoises and Turtles—An International Conference; 1997; New York: Turtle and Tortoise Society and the WCS Turtle Recovery Program.

[53] Decary R. La Fauna malagache. Paris, Payot1950.

[54] Juvik JO, Blanc CP. The Angonoka of Cape Sada. Animals. 1974;16:148–53.

[55] Currylow A. 2016 Baly Bay Ploughshare Tortoise Field Report. 2016 March, 2016.

[56] Currylow AF, Walde AD, Filazaha F, Mandimbihasina A, Woolaver L. Ploughshare Tortoise (*Astrochelys yniphora*) natural entrapment. Herpetology Notes. 2015;8:485–7.

[57] Durrell L, Groombridge B, Tonge S, Bloxam Q, editors. *Geochelone yniphora* ploughshare tortoise, plowshare tortoise, angulated tortoise, angonoka. Broadview, IL: International Union for Conservation of Nature and Natural Resources; 1989.

[58] Gibbons P, Currylow A, Young R, Terry A, Goetz RLM, van Dijk PP, et al. The Ploughshare Tortoise (Astrochelys yniphora): Battle on Multiple Fronts. In: Walde AD, Riedle JD, Lowe H, editors. 14th Annual Symposium on the Conservation and Biology of Tortoises and Freshwater Turtles; August 1–4, 2016; New Orleans, Louisiana, USA: Turtle Survival Alliance; 2016. p. 27.

[59] Morgan J, Chng S. Rising internet-based trade in the Critically Endangered ploughshare tortoise Astrochelys yniphora in Indonesia highlights need for improved enforcement of CITES. Oryx. 2017:1–7.

[60] Kuchling G, Razandrimamilafiniarivo O. The use of ultrasound scanning to study the relationship of vitellogenesis, mating, egg production and follicular atresia in captive Ploughshare Tortoises *Geochelone yniphora*. Dodo. 1999;35:109–15.

[61] Bourou R, Tiandray H, Razandrimamilafiniarivo O, Bekarany E, Durbin J. Comparative reproduction in wild and captive female Ploughshare Tortoises *Geochelone yniphora*. Dodo. 2001;37:70–9.

[62] Miller JD, Dinkelacker SA. Reproductive Structures and Strategies of Turtles In: Wyneken J, Godfrey MH, Bels V, editors. Biology of Turtles. Boca Raton, Florida, USA: CRC Press; 2008.

[63] Currylow AFT, Walker RCJ, Rafeliarisoa TH, Louis EE Jr. Behavior, thermal preference, and ranging patterns of the Critically Endangered Madagascar Spider Tortoise during a cyclone. Herpetol Conserv Biol. 2015;10(2):602–9.

[64] Cagle FR. A system of marking turtles for future identification. Copeia. 1939;1939(3):170–3.

[65] Ernst CH, Hershey MF, Barbour RW. A new coding system for hardshelled turtles. Trans Ky Acad Sci. 1974;35:27–8.

[66] Ferner JW. A Review of Marking and Individual Recognitions Techniques for Amphibians and Reptiles. Moriarty JJ, editor: Society for the Study of Amphibians and Reptiles; 2007 2 2007.

[67] Reid D, Durrell L, Rakotobearison G. The captive breeding project for the angonoka *Geochelone yniphora* in Madagascar. Dodo. 1989;26:34–48.

[68] Hernandez-Divers SM, Hernandez-Divers SJ, Wyneken J. Angiographic, anatomic and clinical technique descriptions of a subcarapacial venipuncture site for Chelonians. J Herpetol Med Surg. 2002;12(2):32–7.

[69] Kuchling G. The Reproductive Biology of the Chelonia. Bradshaw SD, Burggren W, Heller HC, Ishii S, Langer H, Neuweiler G, et al, editors. Berlin: Springer; 1999. 223 p.

[70] Currylow AF, Tift MS, Meyer JL, Crocker DE, Williams RN. Seasonal variations in plasma vitellogenin and sex steroids in male and female Eastern Box Turtles, *Terrapene carolina carolina*. Gen Comp Endocrinol. 2013;180:48–55. doi: 10.1016/j.ygcen.2012.11.005 23174697 10.1016/j.ygcen.2012.11.005

[71] Currylow AFT, Louis EE, Crocker DE. Stress response to handling is short lived but may reflect personalities in a wild, Critically Endangered tortoise species. Conservation Physiology. 2017;5(1):cox008 doi: 10.1093/conphys/cox008 28360999 10.1093/conphys/cox008 PMC5356936

[72] SAS Institute Inc. JMP Pro 12.0.1. Cary, North Carolina, USA: SAS Institute Inc.; 2015.

[73] Slade B, Parrott ML, Paproth A, Magrath MJL, Gillespie GR, Jessop TS. Assortative mating among animals of captive and wild origin following experimental conservation releases. Biol Lett. 2014;10(11):20140656 doi: 10.1098/rsbl.2014.0656 25411380 10.1098/rsbl.2014.0656 PMC4261860

[74] McEwen BS, Wingfield JC. The concept of allostasis in biology and biomedicine. Horm Behav. 2003;43(1):2–15. 12614627 10.1016/s0018-506x(02)00024-7

[75] Baugh AT, Schaper SV, Hau M, Cockrem JF, de Goede P, van Oers K. Corticosterone responses differ between lines of Great Tits (*Parus major*) selected for divergent personalities. Gen Comp Endocrinol. 2012;175(3):488–94. doi: 10.1016/j.ygcen.2011.12.012 22202603 10.1016/j.ygcen.2011.12.012

[76] Fokidis HB, Hurley L, Rogowski C, Sweazea K, Deviche P. Effects of captivity and body condition on plasma corticosterone, locomotor behavior, and plasma metabolites in Curve-Billed Thrashers. Physiological and Biochemical Zoology: Ecological and Evolutionary Approaches. 2011;84(6):595–606. 10.1086/662068 22030852

[77] Dodd CK Jr. North American Box Turtles: A Natural History. Norman, Oklahoma, USA: University of Oklahoma Press; 2001. 231 p.

[78] Ernst CH, Lovich JE. Turtles of the United States and Canada. second ed. Baltimore, Maryland, USA: John Hopkins University Press; 2009.

[79] Riley JL, Tattersall GJ, Litzgus JD. Potential sources of intra-population variation in the overwintering strategy of Painted Turtle (*Chrysemys picta*) hatchlings. J Exp Biol. 2014;217(Pt 23):4174–83. doi: 10.1242/jeb.111120 25324341 10.1242/jeb.111120

[80] Wallace BP, Avens L, Braun-McNeill J, McClellan CM. The diet composition of immature Loggerheads: insights on trophic niche, growth rates, and fisheries interactions. J Exp Mar Biol Ecol. 2009;373(1):50–7.

[81] Jennings AH. Use of habitats and microenvironments by juvenile Florida box turtles, *Terrapene carolina bauri* on Egmont Key. Herpetologica. 2007;63(1):1–10.

[82] Cabezas S, Carrete M, Tella JL, Marchant TA, Bortolotti GR. Differences in acute stress responses between wild-caught and captive-bred birds: a physiological mechanism contributing to current avian invasions? Biol Invasions. 2013;15(3):521–7.

[83] Douxfils J, Mandiki SNM, Marotte G, Wang N, Silvestre F, Milla S, et al Does domestication process affect stress response in juvenile Eurasian perch *Perca fluviatilis*? Comparative Biochemistry and Physiology Part A: Molecular & Integrative Physiology. 2011;159(1):92–9. 10.1016/j.cbpa.2011.01.021 21300167

[84] Thaker M, Lima SL, Hews DK. Acute corticosterone elevation enhances antipredator behaviors in male tree lizard morphs. Horm Behav. 2009;56(1):51–7. doi: 10.1016/j.yhbeh.2009.02.009 19281811 10.1016/j.yhbeh.2009.02.009

[85] Currylow AFT, Louis EE Jr., Crocker DE. Stress response to handling is short-lived, but may reflect personalities in a wild, Critically Endangered tortoise species. Conservation Physiology. in press. 10.1093/conphys/cox008 PMC5356936 28360999

[86] Teixeira CP, de Azevedo CS, Mendl M, Cipreste CF, Young RJ. Revisiting translocation and reintroduction programmes: the importance of considering stress. Anim Behav. 2007;73(1):1–13.

[87] Kelley JL, Magurran AE, Macías-Garcia C. The influence of rearing experience on the behaviour of an endangered Mexican fish, *Skiffia multipunctata*. Biol Conserv. 2005;122(2):223–30.

[88] Pedrono M, Sarovy A. Trial release of the world's rarest tortoise *Geochelone yniphora* in Madagascar. Biol Conserv. 2000;95(3):333–42.

[89] Wallis D. Evaluating the short-term success of a reintroduction of the Critically Endangered Ploughshare tortoise, *Astrochelys yniphora*: Imperial College London; 2009.

[90] Mason GJ. Species differences in responses to captivity: stress, welfare and the comparative method. Trends Ecol Evol. 2010;25(12):713–21. doi: 10.1016/j.tree.2010.08.011 20952089 10.1016/j.tree.2010.08.011

[91] Morales MH, Sánchez EJ. Changes in vitellogenin expression during captivity-induced stress in a tropical anole. Gen Comp Endocrinol. 1996;103(2):209–19. doi: 10.1006/gcen.1996.0112 8812375 10.1006/gcen.1996.0112

[92] Quispe R, Villavicencio CP, Addis E, Wingfield JC, Vasquez RA. Seasonal variations of basal cortisol and high stress response to captivity in *Octodon degus*, a mammalian model species. Gen Comp Endocrinol. 2014;197:65–72. doi: 10.1016/j.ygcen.2013.12.007 24368258 10.1016/j.ygcen.2013.12.007

[93] Callard IP, Callard GV, Lance V, Eccles S. Seasonal changes in testicular structure and function and the effects of gonadotropins in the freshwater turtle, *Chrysemys picta*. Gen Comp Endocrinol. 1976;30(3):347–56. 992355 10.1016/0016-6480(76)90086-1

[94] Licht P. Endocrine patterns in the reproductive cycle of turtles. Herpetologica. 1982;38(1):51–61.

[95] Edwards KL, Shultz S, Pilgrim M, Walker SL. Male reproductive success is correlated with testosterone in the Eastern Black Rhinoceros (*Diceros bicornis michaeli*). Gen Comp Endocrinol. 2015;213:40–9. doi: 10.1016/j.ygcen.2014.12.015 25562628 10.1016/j.ygcen.2014.12.015

[96] Croyle K, Gibbons P, Light C, Goode E, Durrant B, Jensen T. Chelonian perivitelline membrane-bound sperm detection: a new breeding management tool. Zoo Biol. 2016;35:95–103. doi: 10.1002/zoo.21273 26890048 10.1002/zoo.21273

[97] Kempenaers B, Peters A, Foerster K. Sources of individual variation in plasma testosterone levels. Philosophical Transactions of the Royal Society B: Biological Sciences. 2008;363(1497):1711–23. doi: 10.1098/rstb.2007.0001 18048297 10.1098/rstb.2007.0001 PMC2367619

[98] Mendonca MT, Licht P. Seasonal cycles in gonadal activity and plasma gonadotropin in the Musk Turtle, *Sternotherus odoratus*. Gen Comp Endocrinol. 1986;62(3):459–69. 3770437 10.1016/0016-6480(86)90056-0

[99] Wingfield JC, Perfito N, Calisi R, Bentley G, Ubuka T, Mukai M, et al Putting the brakes on reproduction: implications for conservation, global climate change and biomedicine. Gen Comp Endocrinol. 2016;227:16–26. doi: 10.1016/j.ygcen.2015.10.007 26474923 10.1016/j.ygcen.2015.10.007

[100] Currylow AFT, Gibbons P, Goetz M, Walde AD, Louis EE Jr., Crocker DE. Reproductive cycles in wild and captive female Critically Endangered sister tortoises, the Ploughshare (*Astrochelys yniphora*) and Radiated (*A*. *radiata*) of Madagascar. in prep.

[101] Gibbons JW, Lovich JE, Tucker AD, Fitzsimmons NN, Greene JL. Demographic and ecological factors affecting conservation and management of the Diamondback Terrapin (*Malaclemys terrapin*) in South Carolina. Chelonian Conserv Biol. 2001;4(1):66–74.

[102] Hamann M, Godfrey MH, Seminoff JA, Arthur K, Barata PCR, Bjorndal KA, et al Global research priorities for sea turtles: informing management and conservation in the 21st century. Endanger Spec Res. 2010;11(3):245–69.

[103] Wibbels T, Owens DW, Morris YA, Amoss MS, editors. Sexing techniques and sex ratios for immature Loggerhead Sea Turtles captured along the Atlantic Coast of the United States. Ecology of East Florida Sea Turtles: Proceedings of the Cape Canaveral, Florida Sea Turtle Workshop; 1987; Miami, FL: NOAA Technical Report.

[104] Wibbels T, Owens DW, Amoss M. Seasonal changes in the serum testosterone titers of loggerhead sea turtles captured along the Atlantic Coast of the United States. Ecology of East Florida Sea Turtles: Proceedings of the Cape Canaveral, Florida Sea Turtle Workshop. 1987;NMFS 53:58–64.

[105] Rostal DC, Grumbles JS, Lance VA, Spotila JR. Non-lethal sexing techniques for hatchling and immature Desert Tortoises (*Gopherus agassizii*). Herpetol Monogr. 1994:83–7.

[106] Crews D, Cantu AR, Bergeron JM, Rhen T. The relative effectiveness of androstenedione, testosterone, and estrone, precursors to estradiol, in sex reversal in the Red-Eared Slider (*Trachemys-Scripta*), a turtle with temperature-dependent sex determination. Gen Comp Endocrinol. 1995;100(1):119–27. doi: 10.1006/gcen.1995.1140 8575652 10.1006/gcen.1995.1140

[107] Pieau C, Dorizzi M, Richard-Mercier N. Temperature-dependent sex determination and gonadal differentiation in reptiles. Cell Mol Life Sci. 1999;55(6–7):887–900. 10412370 10.1007/s000180050342 PMC11146972

[108] Pedrono M, Smith LL, Sarovy A, Bourou R, Tiandray H. Reproductive ecology of the ploughshare tortoise (*Geochelone yniphora*). J Herpetol. 2001;35(1):151–6.

