# Supplementary material for: Comparative ecophysiology of a critically endangered (CR) ectotherm: Implications for conservation management
Source: PLoS One. 2017 Aug 16;12(8):e0182004. doi: 10.1371/journal.pone.0182004 (PMC5558934; doi:10.1371/journal.pone.0182004)
Supplement: S1 Table — All data underlying the findings described in this manuscript. These are the data points behind means, medians, and variance measures presented in the manuscript by Currylow et al., “Comparative ecophysiology of a Critically Endangered (CR) ectotherm: implications for conservation management”. (PDF) [file pone.0182004.s001.pdf]

Animal_ ID	Sex	Testosterone (ng/dL)	Corticosterone (pg/mL)	Progesterone (ng/mL)	Estradiol (pg/mL)	Population	Month	Ever Sired?	Wt. (g)	SCL (mm)	Activity	°C Air Temp	Ground Temp °C	Ground Humid	Cloud Cover	Veg Cover	Time 2 Blood
80	Female		523.2	1.33	56.72	Wild	April	N	15500	411	Resting in vegetation	29.2	29.3	82.1	0	100	7.44
80	Female		158	0	0	Wild	October	N	12400	414	Resting in vegetation	34.3	35.5	35.4	20	90	0.48
80	Female		154.5	0	68.76	Wild	December	N	11600	410	Resting in vegetation	38.2	38.4	52.9	10	80	1.31
80	Female		193	2.03	39.25	Wild	March	N	13500	413	Resting in vegetation	31	30.7	98	50	70	1.42
81	Sub-adult		1434.2			Wild	April	N	2150	216	Resting in vegetation	33.2	33.1	73.6	20	100	0.34
81	Sub-adult		418.2			Wild	October	N	2500	215	Resting in vegetation	36.1	38.2	30.2	10	80	1.26
81	Sub-adult		960			Wild	December	N	1700	214	Resting in vegetation	34.3	35.2	65.1	70	80	
81	Sub-adult	2.31	420.5			Wild	March	N	2900	231	Resting in open	32.7	34.2	77.2	10	80	1.12
81	Male	68.86	455.9			Wild	March	N	12000	414	Walking	32.6	33.3	87.8	20	80	1.06
82	Male	75.02	192.8			Wild	December	N	11800	440	Resting in vegetation	35.1	37	60.8	20	10	1.59
82	Male	1.69	107.4			Wild	March	N	13400	440	Resting in open				60	90	1.54
83	Female		241.4	1.03	56.15	Wild	December	N	10000	388	Resting in vegetation	33.6	36.2	56.3	40	40	0.5
83	Female		533.6	1.25	26.02	Wild	March	N	11250	388	Resting in open				70	60	1.4
84	Female		291.3	0.61	0	Wild	April	N	12000	390	Resting in open	34.3	34.8	64.6	10	100	2.48
84	Female		233.1	1.01	0	Wild	October	N	10800	390	Resting in vegetation	32.3	35.2	45.6	50	100	0.59
84	Female		412.3	0.51	54.65	Wild	December	N	9500	390	Resting in vegetation	37.1	39	54.5	20	80	1.1
84	Female		326.1	1.68	104.21	Wild	March	N	10750	390	Eating				50	90	
85	Male	522.85	159.9			Wild	December	N	7600	409	Resting in open	30.7	32.1	70	100	90	0.51
85	Male	25.48	70.8			Wild	March	N	9200	406	Eating				20	70	0.45
86	Male	874.02	185.4			Wild	December	N	13000	451	Resting in open	35.2	34.5	55.1	10	50	0.49
86	Male	42	173.2			Wild	March	N	17750	478	Resting in open				80	90	0.54
87	Sub-adult		1243.9			Wild	April	N	86	71	Basking	32.7	33.3	75.6	0	80	0.48
88	Male	0	181.4			Wild	April	N	8500	376	Walking	31.6	32.8	74	50	100	1.2
88	Male	186.27	175.4			Wild	October	N	7500	376	Resting in vegetation	30	32.1	56.3	50	90	0.53
88	Male	38.35	167.3			Wild	December	N	6800	376	Resting in open	32.6	33.4	63.4	50	90	0.47
88	Male	27.1	92.7			Wild	March	N	9750	378	Resting in open				90	90	1.22
89	Female		1136	1.03	39.11	Wild	April	N	13300	398	Resting in vegetation	34.7	36.2	58.3	30	100	75
89	Female		429.2	0.65	32.21	Wild	October	N	10800	397	Resting in open	32.6	35.4	52.4	0	90	2.15
89	Female		119	1.06	60.45	Wild	December	N	8500	394	Resting in vegetation	35.1	38.2	56.5	50	80	1.19
89	Female		290.1	0	97.31	Wild	March	N	11500	397	Resting in open				30	50	
90	Male	1200	308.7			Wild	December	N	12600	459	Resting in open	31	31.4	66.4	90	70	1.25
91	Female		201.8	0	56.34	Wild	December	N	8000	385	Resting in vegetation	37.9	38.2	56.5	30	90	1.14
91	Female		67.3	1.05	0	Wild	March	N	11000	388	Resting in open				10	60	
103	Female		52.9	1.56	184.15	Wild	November	N	10800	383	Resting in open	35.4	37.3	72.1	20	60	0.48
104	Male	35.41	74.9			Wild	April	N	18750	468	Resting in vegetation	27.5	24.2	84.3	100	100	0.39
104	Male	1200	220.7			Wild	October	N	17900	469	Resting in vegetation	33.4	33.6	56.1	0	60	0.39
104	Male	1200	1126.3			Wild	November	N	15200	467	Resting in open	34.6	42.5	58.4	30	40	2.08
105	Male	0	86.2			Wild	April	N	13000	431	Resting in vegetation	31.8	29.6	88	80	100	0.44
105	Male	1200	212.6			Wild	October	N	12300	434	Resting in vegetation	33.2	33.5	59	30	70	0.36
105	Male	1200	289.9			Wild	November	N	12000	433	Resting in open	32.1	34.2	73.5	50	100	0.5
106	Female		43.9	0.99	0	Wild	April	N	11000	384	Resting in vegetation	30.9	31.3	60.6	70	90	1.1
107	Female		5000	1.5	30.62	Wild	April	N	11250	393	Resting in vegetation	30	32.5	79.4	90	100	28.54
107	Female		2449	0	47.92	Wild	October	N	11600	392	Resting in open	33	33.6	65.5	20	80	1.06
107	Female		1428.6	0.93	0	Wild	November	N			Resting in open				100	60	
107	Female		2683.2	0.68	40.11	Wild	November	N	12000	388	Eating	34	34.8	58.6	30	60	2.2
108	Male	0	52.8			Wild	April	N	13250	432	Resting in vegetation	30.3	30.1	70.6	10	100	3
108	Male	1200	181.6			Wild	October	N	12600	433	Resting in open	34	35.9	57.3	20	70	2.27
108	Male	722.16	651			Wild	November	N	12800	430	Resting in vegetation	30	25.6	88.7	100	10	0.46
109	Female		298.3	0.92	0	Wild	April	N	11750	403	Basking	27	27.5	85.9	30	60	4.5
109	Female		232.5	0	0	Wild	October	N	10600	403	Walking	31.5	32.7	59.9	40	70	0.47
109	Female		123.5	1.22	81.56	Wild	November	N	11250	402	Resting in open	29.8	30.4	89.3	30	40	0.43
110	Male	1200	166.5			Wild	November	N	11800	435	Walking	31	31.9	76.8	10	20	0.34
111	Male	1200	232.5			Wild	November	N	12000	445	Resting in open	35.2	38.1	71.2	30	10	0.29
112	Male	1200	242.8			Wild	November	N	12000	431	Resting in open	31	35.2	58.2	100	50	
113	Female		77.3	1.14	0	Wild	April	N	12250	395	Resting in open	30.5	32	66	90	100	1.32
113	Female		248.5	0.52	25.79	Wild	October	N	10800	395	Resting in vegetation	32.6	34.9	55.7	40	70	3.3
113	Female		86.1	1.99	118.69	Wild	November	N	11200	392	Walking	32.9	33.2	68.8	20	60	0.4
114	Sub-adult		211.2			Wild	November	N	7000	325	Resting in open	31.3	31.8	88.7	30	50	
115	Male	1200	668.3			Wild	November	N	11200	416	Resting in open	33.9	34.6	75.2	20	40	1.47
116	Male	87.78	75			Wild	April	N		519	Resting in open	36.7	34.8	67.4	20	100	4.54
116	Male	1200	103.5			Wild	October	N	17000	519	Resting in vegetation	34.1	34.5	41.4	100	90	0.57
116	Male	1007.85	274.1			Wild	December	N	14400	519	Resting in open	22.3	25.1	60.7	0	100	0.34
116	Male	311.38	34.1			Wild	March	N	18750	519	Resting in open	26.8	27.3	100	100	100	1.11
117	Male	1164.77	345			Wild	December	N	11900	467	Resting in open	32.9	35.8	52.7	50	50	1.06
118	Male	42.74	128.4			Wild	April	N		470	Resting in vegetation	35.7	37.6	70.2	50	10	1.38
118	Male	1200	135.7			Wild	October	N	17300	470	Resting in open	29.6	30.7	41.5	90	40	0.44
118	Male	1200	233.2			Wild	December	N	13750	466	Resting in open	35	35.5	51.3	0	60	0.38
118	Male	55.49	282.6			Wild	March	N	16200	472	Resting in open	28.1	28	97.7	100	100	0.38
119	Sub-adult		1143.3			Wild	April	N	900	151	Resting in vegetation	33	33.8	73.5	0	100	2
119	Sub-adult		1983.6			Wild	October	N	850	150	Resting in vegetation	32.9	35.4	51.4	70	80	4
119	Sub-adult		2774.6			Wild	December	N	860	153	Walking	32.8	36	61.6	90	80	2.08
120	Female		208.4	14.63	135.13	Wild	February	N	8400	375	Unrecorded	28.9	29.6	88	90	20	
121	Female		960.1	2.21	115.34	Wild	February	N	8000	358	Eating	29.6	33.4	79.5	90	10	
122	Female		928.2	1.9	175.86	Wild	January	N	7500	332	Resting in open	32.6	34	87.6	60	50	2.2
122	Female		990.6	0.8	0	Wild	May	N	6250	338	Resting in open	31.5	35.1	53.3	10	60	2.14
122	Female		2597.9	0	0	Wild	October	N	5600	333	Resting in vegetation	30.6	33	60.6	10	40	4.3

122	Female		276.4	3.45	0	Wild	November	N	7000	333	Resting in open						
122	Female		496.8	4.36	194.08	Wild	February	N	6500	338	Walking	27.4	29.2	86.7	100	50	
123	Female		430	2.48	0	Wild	October	N	10200	404	Resting in vegetation	29.4	29.9	64	10	80	1.26
123	Female		836.6	0	0	Wild	November	N	11000	403	Resting in vegetation						1.59
123	Female		423.4	2.33	0	Wild	November	N			Resting in vegetation						0.36
123	Female		269.2	1.68	0	Wild	February	N	10750	407	Resting in vegetation	33.8	34.4	93	20	40	0.48
124	Male	185.45	223.2			Wild	January	N	11200	436	Eating	32.6	35.7	73.9	10	60	1
124	Male	22.79	596.7			Wild	May	N	11250	430	Resting in vegetation	33.4	35.3	61.9	10	60	0.47
124	Male	1200	520.8			Wild	October	N	10800	436	Resting in vegetation	28.9	29	68.8	10	80	1.26
124	Male	1200	453.7			Wild	November	N	9800	432	Resting in open						
124	Male	1200	463.4			Wild	November	N			Resting in open						1.04
125	Female		603.7	2.46	114.41	Wild	January	N	7200	354	Resting in open	34.3	34.1	76	50	30	0.41
125	Female		400.5	1.25	83.15	Wild	February	N	7800	354	Resting in open	33.7	34.3	77.3	20	10	1
126	Female		750	1.53	124.94	Wild	February	N	8600	362	Resting in open	30	31.1	86.8	90	20	0.46
127	Female		9845.9	14.62	85.61	Wild	January	N	7900	364	Resting in open	32.9	34.2	70.7	70	30	0.3
127	Female		4348.4	0.83	0	Wild	May	N	7750	368	Resting in open	30.5	31.5	63.6	10	30	0.4
127	Female		2575.8	0	32.27	Wild	October	N	7500	366	Resting in vegetation	29.8	31.3	64.8	10	80	1.47
127	Female		2785.9	1.31	135.34	Wild	November	N	8500	366	Resting in open	33.6	37.1	73	10	50	0.32
128	Female		1422.5	0.94	153.13	Wild	January	N	7400	361	Resting in open	32.9	34.2	76.1	30	60	2.3
128	Female		784.8	0.6	0	Wild	May	N	7000	361	Resting in open	30	30.4	65.8	90	100	0.31
128	Female		903.6	0	0	Wild	October	N	6000	361	Resting in vegetation	31.8	34	62.6	10	60	4.52
128	Female		708	1.15	59.88	Wild	November	N	7250	360	Resting in open	34.4	35.3	70	10	50	2.2
128	Female		1176.5	0.96	124.02	Wild	February	N	7500	361	Resting in vegetation	32.8	34.9	80.9	30	30	
129	Female		1891.4	1.88	24.25	Wild	January	N	8000	371	Resting in open	31.3	32.3	85.5	80	10	0.5
130	Female		5128	1.43	43.42	Wild	May	N	9300	398	Resting in open	33.8	34.8	59.7	50	100	36.15
131	Female		83.4	0.92	59.39	Wild	November	N	12200		Resting in open	32.2	33.2	78.7	80	80	0.54
132	Male	1081.7	3703.5			Wild	November	N	10800	416	Eating	29.4	31.9	69.3	100	60	1.2
133	Male	543.3	594.3			Wild	January	N	9800	419	Eating	31.5	33.4	77.7	20	30	0.55
133	Male	1200	842.4			Wild	November	N	10200	419	Resting in open	31.9	34.2	58	20	20	1.08
133	Male	441.5	436.6			Wild	February	N	10200	416	Resting in open	30.5	35.2	75.1	10	50	
134	Female		31641.3	0.69	30.73	Wild	February	N	9400	393	Resting in open	33.8	34.1	86	10	50	
135	Male	0	257.1			Wild	May	N	9500	411	Resting in vegetation	32.1	31.4	63.1	0	50	
135	Male	1200	1332.2			Wild	October	N	8250	413	Resting in vegetation	33.4	35.7	60	10	50	2.14
135	Male	1200	3183.2			Wild	November	N	8500	413	Eating						
135	Male	484.8	783.1			Wild	February	N	9500	413	Resting in open	31.7	32.7	99.8	10	50	
136	Female		458.2	0.75	120.37	Wild	February	N			Walking						35.07
137	Male	65.96	119.8			Wild	February	N	11750	445	Resting in open						0.55
138	Male	140.78	445.1			Wild	May	N	14000	456	Resting in open	31.6	32.3	69.6	30	90	4.4
138	Male	1036.54	348.7			Wild	October	N	11600	453	Resting in vegetation	30.7	33.7	65.5	0	50	1.1
138	Male	1200	910.3			Wild	November	N	12000	453	Resting in open	31.1	31.7	81.5	90	80	0.58
138	Male	67.7	580.8			Wild	February	N	12000	445	Resting in open	33.5	35.1	84.4	50	100	0.25
139	Female		1047.1	1.72	165.09	Wild	January	N	8500	384	Walking	30.6	32.8	73.8	30	80	0.51
139	Female		582	1.14	0	Wild	November	N	9000	385	Resting in open	28.1	29.2	76.1	90	20	0.22
139	Female		403.5	14.18	163.56	Wild	February	N	8000	383	Resting in vegetation	32	32.7	100	40	30	0.47
140	Female		2260.1	5.31	198.25	Wild	February	N	8600	389	Reproductive/Nesting						
141	Female		2594.1	1.77	123.59	Wild	November	N	7600		Resting in open	33.5	37.6	74.2	10	40	
142	Male	1016.41	15877.1			Wild	November	N	12100	444	Resting in open						1.5
142	Male	97.24	5000			Wild	February	N	13500	445	Walking	33.4	35.3	86.2	40	80	1.55
143	Female		404.1	0.5	122.63	Wild	October	N	9200	388	Resting in open	29.1	31.9	70.4	10	60	1.06
143	Female		190.2	1.56	179.97	Wild	November	N	10100	384	Resting in vegetation	31.6	33.5	74.2	50	40	0.46
144	Male	217.13	130.2			Wild	January	N	8600	394	Walking	34.5	34.7	79.6	50	80	1.1
145	Female		499.5	1.14	36.4	Wild	May	N	10000	401	Resting in open	35.3	35.6	49.2		90	0.5
146	Male	318.45	330.1			Wild	January	N	12000	449	Resting in open	30.4	32.9	86.5	10	80	1.41
146	Male	67.86	295.5			Wild	May	N	13000	449	Resting in open	34.5	35	55.2	0	100	0.4
146	Male	1200	1114.6			Wild	October	N	11800	449	Resting in vegetation	34.4	35.8	58.6	30	50	1.3
146	Male	1107.93	647.1			Wild	November	N	11600	449	Walking						2.1
146	Male	188.06	2935.7			Wild	February	N	12400	449	Resting in vegetation	27.1	26	97.6	20	40	43
147	Male	1200	496.3			Wild	November	N	11600	432	Walking						0.5
148	Sub-adult		6419.2			Wild	January	N	1500	167	Resting in open	31.4	34.7	69.7	60	60	12
148	Sub-adult		26971.1			Wild	May	N	1100	171	Resting in vegetation	33	38.8	51.1	10	70	10.15
148	Sub-adult		12115.7			Wild	October	N	1000	170	Resting in vegetation	30.9	36.8	52.6	10	40	8.2
148	Sub-adult		3799.8			Wild	November	N	1050	172	Resting in vegetation	33.6	40	53	10	40	2.56
149	Sub-adult		8094.4			Wild	January	N	331	110	Resting in open	29	31.1	82.2	20	20	1.3
150	Sub-adult	0	1140.1			Wild	February	N	341	126	Resting in open	31.7	38.2	79.9	10	40	
151	Sub-adult	39.29	1284.6			Wild	February	N	155	90	Resting in vegetation				10	10	
1	Female		691.9	1.09	0	Madagascar Captive	January	Y	10800	349	Resting in open	35.2			0	30	0.42
1	Female		898.9	0.44	91.67	Madagascar Captive	November	Y	10600	396	Resting in open	31.7	32.6	63.8	60	10	2.02
1	Female		352.9	0	39.31	Madagascar Captive	July	Y	12000	392	Resting in open	27.3	27.2	65.6	80	20	
1	Female		292.1	0.45	0	Madagascar Captive	March	Y	11400	396	Walking				0		0.3
2	Male		304.4			Madagascar Captive	July	Y	11600	438	Resting in open	27.3	27.2	65.6	80	20	
2	Male	27.9	462.8			Madagascar Captive	March	Y	10400	440	Walking				0		0.43
3	Male	48.19	901.5			Madagascar Captive	July	Y	17200	486	Resting in open	27.3	27.2	65.6	80	20	
3	Male	13.18	928.6			Madagascar Captive	March	Y	15200	487	Walking				0		
4	Male	53.27	315			Madagascar Captive	January	Y	11100	419	Resting in vegetation	35.4			0	30	2.29
4	Male	241.8	32			Madagascar Captive	July	Y	12400	419	Resting in open	27.3	27.2	65.6	80	20	
4	Male	23.91	91			Madagascar Captive	March	Y	11400	422	Walking		34.7	65.5	10		0.39
5	Male	184.11	34.3			Madagascar Captive	July	Y	13200	427	Resting in open	27.3	27.2	65.6	80	20	
5	Male	61.36	260.9			Madagascar Captive	March	Y	11200	430	Walking				10		0.26

6	Female	0	651.3			Madagascar	Captive	January	N	8000	359	Unrecorded						
6	Female		65.3	0	0	Madagascar	Captive	May	N	8800	364	Resting in open	31.7	31.6	63.7	0	80	0.34
6	Female		166.9	0	0	Madagascar	Captive	September	N	7500	363	Resting in open				40	60	4.54
6	Female		372.5	0.7	0	Madagascar	Captive	September	N	7500	363	Resting in open				40	60	4.54
6	Female		10	0.57	0	Madagascar	Captive	June	N	7800	362	Unrecorded	26.4	26.4	53.9			0.48
7	Female		261.4	0.87	0	Madagascar	Captive	January	N	11200	402	Eating	35.1			0	30	5
8	Female		224.4	0	0	Madagascar	Captive	November	Y	14400	403	Resting in open	31.7	32.6	63.8	60	10	0.23
8	Female		559.7	0.72	0	Madagascar	Captive	July	Y	12200	395	Resting in open	27.3	27.2	65.6	80	20	
8	Female		497.4	0.82	0	Madagascar	Captive	March	Y	11400	404	Walking				0		0.54
9	Sub-adult	0	457.9			Madagascar	Captive	March	N	2600	238	Walking				0		0.49
10	Male	128.43	69.4			Madagascar	Captive	July	N	12200	422	Resting in open	27.3	27.2	65.6	80	20	
10	Male	27.12	232.7			Madagascar	Captive	March	N	12400	421	Walking				0		0.31
11	Sub-adult	0	79.7			Madagascar	Captive	March	N	4200	274	Walking						1.25
12	Female		240.7	0	61.05	Madagascar	Captive	July	N	7900	342	Resting in open	27.3	27.2	65.6	80	20	
12	Female		450.3	0.86	0	Madagascar	Captive	March	N	7600	344	Walking						0.3
13	Sub-adult		7273.3			Madagascar	Captive	January	N	2390	234	Eating				0	20	0.37
13	Sub-adult	0	24381.1			Madagascar	Captive	March	N	3400	263	Walking	30.1	29.7	68	0		1.43
14	Female		2631.1	2.42	86.53	Madagascar	Captive	January	Y	10400	380	Resting in open				60	80	1.21
14	Female		4045.4	2.22	135.3	Madagascar	Captive	November	Y	9500	376	Resting in open	32.7	32.6	67.5	60	20	0.36
14	Female		1044.7	0	0	Madagascar	Captive	July	Y	10800	380	Resting in open	27.3	27.2	65.6	80	20	
14	Female		3770.7	1.06	0	Madagascar	Captive	March	Y	9800	382	Walking						0.3
15	Sub-adult	0	370.9			Madagascar	Captive	March	N	4000	268	Walking				0		0.27
16	Sub-adult	1.56	3433.8			Madagascar	Captive	March	N	4600	294	Walking	31.5	30.7	64.2			1.17
17	Male	42.66	414.3			Madagascar	Captive	January	Y	14600	479	Unrecorded						
17	Male	4.55	350.1			Madagascar	Captive	May	Y	16000	480	Walking	28.5	28.8	73.7	70	50	1
17	Male	386.6	742.4			Madagascar	Captive	September	Y	14000	478	Eating				0	10	6.1
17	Male	1200	1640.5			Madagascar	Captive	November	Y	14000	478	Resting in open	30.2	31	70.3	80	20	1.01
17	Male	24.23	1409.5			Madagascar	Captive	June	Y	15200	480	Unrecorded	26.8	24.6	57.9			2
17	Male	45.87	230.3			Madagascar	Captive	February	Y	14800	480	Eating	30.3	30.9	80.8	100	30	1.23
17	Male	2.83	167.2			Madagascar	Captive	March	Y	14000	481	Unrecorded	26.9	27.8	75.4	0		
18	Male	41.1	739			Madagascar	Captive	January	Y	13600	460	Unrecorded						
18	Male	40.69	1160.2			Madagascar	Captive	May	Y	14600	465	Resting in open	30.2	29.2	74.3	10	60	3
18	Male	300.54	494.4			Madagascar	Captive	September	Y	15000	462	Resting in open				40	20	0.41
18	Male	578.63	1420.6			Madagascar	Captive	November	Y	13600	460	Resting in open	33	32.4	67	50	70	2.43
18	Male	20.68	669.6			Madagascar	Captive	February	Y	14400	463	Resting in open	26.8	27.8	84.2	100	20	
19	Male	0	206.7			Madagascar	Captive	January	Y	12400	424	Unrecorded						
19	Male	709.15	2157.1			Madagascar	Captive	September	Y	13000	424	Eating				10	40	0.54
19	Male	661.53	2647.4			Madagascar	Captive	November	Y	11600	424	Unrecorded	30.6	31.3	73.3	80	50	0.35
19	Male	6.27	1987.8			Madagascar	Captive	June	Y		422	Unrecorded	26.8	24.6	57.9			1.14
19	Male	23.47	360.4			Madagascar	Captive	February	Y	12400	425	Walking	30.4	33.6	75.9	100	80	0.32
20	Male	45.29	6004.6			Madagascar	Captive	January	Y	11000	405	Unrecorded						
20	Male	9.72	5117.1			Madagascar	Captive	May	Y	11400	408	Walking	30.2	29.2	74.3	10	60	0.29
20	Male	1200	4199.1			Madagascar	Captive	September	Y	11800	407	Resting in open				50	10	1.24
20	Male	371.4	3707.2			Madagascar	Captive	November	Y	11200	407	Resting in open	33	32.4	67	50	50	0.53
20	Male	160.18	2684.2			Madagascar	Captive	June	Y	12200	404	Unrecorded	27.5	26.9	49.5			0.25
20	Male	21.54	7269.8			Madagascar	Captive	February	Y	11200	406	Walking	26.8	27.8	84.2	100	20	0.19
21	Male	10.8	806.3			Madagascar	Captive	January	N	12400	419	Unrecorded						
21	Male	2.22	344.9			Madagascar	Captive	May	N	13400	424	Walking	28.5	28.8	73.7	40	50	0.34
21	Male	723.2	496.3			Madagascar	Captive	September	N	12800	423	Eating				10	50	0.28
21	Male	739.6	576.3			Madagascar	Captive	November	N	11600	424	Unrecorded	30.6	31.3	73.3	80	50	0.39
21	Male	112.23	219.1			Madagascar	Captive	June	N	14000	423	Unrecorded	26.8	24.6	57.9			0.27
21	Male	19.29	323.4			Madagascar	Captive	February	N	13200	428	Resting in open	30.4	33.6	75.9	100	80	0.25
21	Male	4.51	249.1			Madagascar	Captive	March	N	12300	424	Unrecorded	27.1	26.7	80.5	0		0.53
22	Male	44.93	238.4			Madagascar	Captive	January	Y	14400	457	Unrecorded						
22	Male	8.99	185			Madagascar	Captive	May	Y	14200	456	Walking	28.5	28.8	73.7	70	50	0.49
22	Male	917.09	136.5			Madagascar	Captive	September	Y	14600	455	Resting in open				10	40	0.57
22	Male	1200	295.1			Madagascar	Captive	November	Y	12400	455	Unrecorded	30.2	31	70.3	80	20	1.09
22	Male	8.66	119.2			Madagascar	Captive	June	Y	14600	453	Unrecorded	26.8	24.6	57.9			1.04
22	Male	0	50.8			Madagascar	Captive	February	Y	14200	456	Walking	30.4	33.6	75.9	100	80	0.27
22	Male	9.5	199.1			Madagascar	Captive	March	Y	13100	455	Unrecorded	26.9	27.8	75.4	0		
23	Male		540.6	1.15	0	Madagascar	Captive	January	Y	9800	417	Unrecorded						
23	Male	4.32	200.9			Madagascar	Captive	May	Y	10300	418	Walking	28.5	28.8	73.7	70	50	0.42
23	Male	157.21	315.8			Madagascar	Captive	September	Y	10000	416	Eating				0	50	4.17
23	Male	266.8	134.9			Madagascar	Captive	November	Y	10100	416	Unrecorded	30.2	31	70.3	80	20	0.29
23	Male	3.01	70.5			Madagascar	Captive	June	Y	11400	409	Unrecorded	26.8	24.6	57.9			0.38
23	Male	5.46	62.6			Madagascar	Captive	February	Y	11000	416	Eating	30.3	30.9	80.8	100	30	0.27
23	Male	21.54	118.6			Madagascar	Captive	March	Y	11000	415	Resting in open	26.9	27.8	75.4	0		0.32
24	Male	58.27	246			Madagascar	Captive	January	N	10800	420	Unrecorded						
24	Male	10.87	109.8			Madagascar	Captive	May	N	12000	420	Walking	30.2	29.2	74.3	0	60	1.29
24	Male	1200	74.5			Madagascar	Captive	September	N	12000	418	Resting in open				50	10	3.03
24	Male	618.72	152.3			Madagascar	Captive	November	N	10800	419	Resting in open	33	32.4	67	50	20	0.29
24	Male	41.74	81.4			Madagascar	Captive	July	N	12000	418	Resting in open	28.9	28.4	49.5	80	20	0.45
24	Male	35.98	141.2			Madagascar	Captive	February	N	11700	420	Walking	26.8	27.8	84.2	100	20	2.15
24	Male	13.6	401.3			Madagascar	Captive	March	N	12200	426	Unrecorded	26.9	27.8	75.4	0		2
25	Female		214.5	1.55	89.53	Madagascar	Captive	January	Y	10800	393	Unrecorded						
25	Female		138.4	1.05	48.72	Madagascar	Captive	May	Y	11800	398	Walking	31.7	31.6	63.7	0	80	0.29
25	Female		229.2	0	35.13	Madagascar	Captive	September	Y	11900	393	Walking				10	60	1
25	Female		140.1	0.47	0	Madagascar	Captive	November	Y	11000	397	Unrecorded	30.8	31.6	66.6	70		0.43

25	Female		75.2	0.72	50.47	Madagascar	Captive	June	Y	11800	397	Unrecorded	26.4	26.4	53.9			0.32
25	Female		36.1	0	0	Madagascar	Captive	February	Y	11100	398	Walking	26.8	27.7	89.9	100	30	0.38
26	Female		99.5	0.48	0	Madagascar	Captive	September	Y	11800	394	Resting in open				40	60	4.02
26	Female		208.9	0.62	185.11	Madagascar	Captive	November	Y	11800	392	Resting in open	30.8	31.6	66.6	50	40	1.09
26	Female		10	0.45	0	Madagascar	Captive	June	Y	11400	395	Unrecorded	26.4	26.4	53.9			2.14
26	Female		29.2	0.77	0	Madagascar	Captive	February	Y	11000	394	Resting in open	26.8	27.7	89.9	100	30	2.4
27	Male	9.9	15977.4			Madagascar	Captive	January	Y	10600	421	Unrecorded						
27	Male	4.27	2988			Madagascar	Captive	May	Y	11900	421	Eating	28.5	28.8	73.7	60	50	0.34
27	Male	176.68	5940.7			Madagascar	Captive	September	Y	10700	420	Eating				0	30	3.45
27	Male	1200	4865.9			Madagascar	Captive	November	Y	10800	420	Unrecorded	30.6	31.3	73.3	80	20	0.27
27	Male	12.83	5000			Madagascar	Captive	June	Y	12400	420	Unrecorded	26.8	24.6	57.9			0.45
27	Male	15.97	6274.7			Madagascar	Captive	February	Y	11500	421	Eating	30.3	30.9	80.8	100	30	1.25
27	Male	34.18	7509.6			Madagascar	Captive	March	Y	10300	423	Unrecorded	26.9	27.8	75.4	0		0.36
28	Female		762.8	1.35	49.97	Madagascar	Captive	January	Y	11600	414	Unrecorded						
28	Female		524.2	0	0	Madagascar	Captive	May	Y	12800	416	Resting in open	31.7	31.6	63.7	0	80	
28	Female		1005.2	0.6	73.48	Madagascar	Captive	November	Y	12000	415	Resting in open	30.8	31.6	66.6	50	40	0.29
28	Female		296.3	0	0	Madagascar	Captive	July	Y	12600	413	Resting in open	28.9	28.4	49.5	80	30	0.25
28	Female		453.3	0.61	89.47	Madagascar	Captive	February	Y	12200	415	Unrecorded	26.8	27.7	89.9	100	30	0.49
29	Female		47.4	0.42	0	Madagascar	Captive	May	N	11100	379	Resting in open	31.7	31.6	63.7	0	80	0.36
29	Female		176.9	0.46	111.92	Madagascar	Captive	September	N	10000	377	Resting in open				40	60	8.4
29	Female		405.1	0.81	104.43	Madagascar	Captive	November	N	10000	378	Unrecorded	30.8	31.6	66.6	70		1.02
29	Female		10	0.75	72.76	Madagascar	Captive	June	N	10600	378	Unrecorded	26.4	26.4	53.9			0.55
29	Female		40.5	0	40.87	Madagascar	Captive	February	N	9200	376	Walking	26.8	21.7	89.1	100	30	1.38
30	Female	20.77	133.9			Madagascar	Captive	January	Y	9700	372	Unrecorded						
30	Female		67.4	1.12	0	Madagascar	Captive	May	Y	10600	373	Walking	31.7	31.6	63.7	0	80	0.29
30	Female		157.7	0.49	33.02	Madagascar	Captive	September	Y	10000	372	Resting in open				50	60	1
30	Female		465.6	1	89.66	Madagascar	Captive	November	Y	9800	372	Resting in open	30.8	31.6	66.6	60	40	0.49
30	Female		31.8	0.43	0	Madagascar	Captive	June	Y	10200	371	Unrecorded	26.4	26.4	53.9			1
30	Female		55.2	0.58	0	Madagascar	Captive	February	Y	9400	373	Walking	26.8	27.7	89.9	100	30	2.28
31	Female		76.2	1.38	58.64	Madagascar	Captive	May	Y	14000	410	Resting in open	31.7	31.6	63.7	0	80	1.09
31	Female		100.6	0.61	117.77	Madagascar	Captive	September	Y	14000	409	Walking				10	60	0.28
31	Female		169.7	1.95	109.8	Madagascar	Captive	November	Y	12800	409	Resting in open	30.8	31.6	66.6	50	40	0.59
31	Female		10	0.42	42.18	Madagascar	Captive	June	Y	13000	409	Unrecorded	26.4	26.4	53.9			0.2
31	Female		29.4	0.86	62.68	Madagascar	Captive	July	Y	13000	409	Resting in vegetation	28.9	28.4	49.5	80	30	0.48
31	Female		10	0.51	57.7	Madagascar	Captive	February	Y	11600	411	Walking	26.8	27.7	89.9	100	30	0.3
31	Female		75.5	1.15	27.09	Madagascar	Captive	March	Y	13000	409	Unrecorded	29.3	28.3	75.4	0		0.27
32	Male	123.46	3497.1			Madagascar	Captive	January	Y	11600	433	Unrecorded						
32	Male	144.54	1732.9			Madagascar	Captive	May	Y	13300	436	Walking	30.2	29.2	74.3	0	60	1.19
32	Male	1200	2643			Madagascar	Captive	September	Y	12000	434	Resting in open				50	10	3.03

32	Male	1109.26	2169.7			Madagascar	Captive	November	Y	12000	435	Resting in open	33	32.4	67	50	100	10.9
32	Male	74.68	2459.1			Madagascar	Captive	February	Y	12400	438	Walking	26.8	27.7	89.9	100	30	
33	Female		321.6	1.46	42.26	Madagascar	Captive	January	Y	9400	372	Unrecorded						
33	Female		257.4	0.66	0	Madagascar	Captive	September	Y	10000	372	Walking				40	60	0.3
33	Female		819.8	0.9	124.06	Madagascar	Captive	November	Y	9800	372	Resting in open	30.8	31.6	66.6	50	40	0.33
33	Female		179.3	0.64	24.86	Madagascar	Captive	June	Y	10000	374	Unrecorded	26.4	26.4	53.9			0.45
33	Female		483.7	1.1	0	Madagascar	Captive	March	Y	8400	372	Unrecorded	29.3	28.3	75.4	0		0.35
35	Female		879.7	1.39	95.23	Madagascar	Captive	January	N	7300	345	Unrecorded						
35	Female		467.3	0.95	0	Madagascar	Captive	May	N	8000	348	Walking	32.3	31.6	64.5	0	60	1.07
35	Female		503.6	0	74.83	Madagascar	Captive	September	N	7800	347	Walking				10	10	2.56
35	Female		298.8			Madagascar	Captive	December	N	8000	350	Resting in open	26.8	26.8	93.8	100	80	1.29
36	Female		1253	1.59	37.1	Madagascar	Captive	January	N	8300	350	Unrecorded						
36	Female		1635.2	0.67	0	Madagascar	Captive	May	N	9600	355	Resting in open	32.3	31.6	64.5	0	60	1.3
36	Female		3583.1	0.57	61.54	Madagascar	Captive	September	N	9100	355	Walking				10	40	1.03
36	Female		1651	0	26.67	Madagascar	Captive	December	N	9250	357	Resting in open	26.8	26.8	93.8	100	80	0.32
36	Female		1154.4	0.44	0	Madagascar	Captive	July	N	9400	356	Resting in open	28.9	28.4	49.5	80	30	1.58
37	Female		621.3	0.76	44.57	Madagascar	Captive	January	N	7400	322	Unrecorded						
37	Female		512.1	0.79	69.85	Madagascar	Captive	September	N	7600	321	Eating				10	10	2.2
37	Female		726.2			Madagascar	Captive	December	N	7600	325	Walking	26.6	26.9	94.6	100	40	1.37
37	Female		239.1	1.02	61.25	Madagascar	Captive	February	N	9000	332	Walking	30.8	31.6	77.7	70	70	1.19
37	Female		534.8	0.77	0	Madagascar	Captive	March	N	7400	333	Unrecorded	27.1	26.7	80.5	0		0.26
38	Female		11528.9	1.65	91.29	Madagascar	Captive	January	N	6900	330	Unrecorded						
38	Female		1660.8			Madagascar	Captive	December	N	7000	332	Resting in open	26.8	26.8	93.8	100	80	0.5
38	Female		448.3	0	0	Madagascar	Captive	May	N	6800	335	Resting in open	31.9	32.1	74.8	0	60	0.29
38	Female		271.8			Madagascar	Captive	December	N	6700	338	Walking	27.3	28.1	88.6	100	40	0.3
38	Female		571.3	0.61	0	Madagascar	Captive	March	N	6300	341	Unrecorded	29.3	28.3	75.4	0		1.15
39	Female		156.5	0.59	0	Madagascar	Captive	May	N	8100	349	Resting in open	31.9	32.1	74.8	0	60	1.01
39	Female		191.6			Madagascar	Captive	December	N	8200	351	Walking	27.3	28.1	88.6	100	40	0.5
39	Female		95	0	0	Madagascar	Captive	February	N	8900	359	Eating	30.7	30.9	80.2	70	70	0.41
39	Female		69.7	0.61	0	Madagascar	Captive	March	N	9000	360	Unrecorded	27.1	26.7	80.5	0		
40	Sub-adult		216.6	0.64	0	Madagascar	Captive	May	N	5200	294	Resting in open	31.4	29.8	68.7	0	60	
40	Sub-adult		232.3			Madagascar	Captive	December	N	5800	297	Walking	26.7	27.3	89.5	100	40	1.06
40	Sub-adult		133.4	0	0	Madagascar	Captive	February	N	6500	307	Resting in open	30.7	30.9	80.2	70	70	0.22
41	Sub-adult		71.7	0.52	0	Madagascar	Captive	May	N	4800	289	Resting in open	31.4	29.8	68.7	0	60	1.1
41	Sub-adult		78.4			Madagascar	Captive	December	N	5000	295	Resting in open	26.7	27.3	89.5	100	40	0.47
42	Female		2709.2	0.65	0	Madagascar	Captive	May	N	7600	333	Resting in open	32.3	31.6	64.5	0	60	0.42
42	Female		4461	0	0	Madagascar	Captive	September	N	7400	332	Walking				10	10	1.37
42	Female		2410.2			Madagascar	Captive	December	N	7600	338	Eating	26.6	26.9	94.6	100	40	0.38
42	Female		1885.6	0.58	42.24	Madagascar	Captive	February	N	9000	348	Walking	30.8	31.6	77.7	70	70	0.48

76	Sub-adult		305.3			Madagascar Captive	November	N	3000	237	Resting in open	35.6	35.4	53.2	70	80	1.3
77	Sub-adult		462.6			Madagascar Captive	May	N	1150	171	Eating	30.9	31	67	0	50	2.25
78	Sub-adult		482.3			Madagascar Captive	May	N	1100	166	Eating	30.9	31	67	0	50	2.19
79	Sub-adult	23.57	527.1			Madagascar Captive	March	N	6600	323	Walking	32.1			0		2.15
92	Female		12271.9	2.03	71.11	US Captive	July	Y	10640	402	Resting in open	30.2	30.2		0	60	1.4
92	Female		5261.1	1.59	76.33	US Captive	August	Y	11590	387	Resting in open	37.2	35.1	40	0	40	
92	Female		5419.6	1.12	54.86	US Captive	December	Y	10710	385	Walking	32	33.1				0.32
92	Female		3903.7	1.08	48.81	US Captive	January	Y	11410	388	Eating	25.6	25.4	27.3	30		0.44
92	Female		3527.6	1.12	45.31	US Captive	February	Y	10430	387	Resting in vegetation	29.6	28.5	62.5			1.48
92	Female	36.03	5000	2.4	139.34	US Captive	September	Y	11340	387	Unrecorded				0	30	
92	Female		4653.6	5.59	66.19	US Captive	October	Y	10880	387	Basking	24.8	24.8	59.9	0	60	
92	Female		5000	1.55	48.92	US Captive	November	Y	12310	387	Walking						
92	Female		19023	1.01	91.34	US Captive	December	Y	11890	387	Resting in open						
92	Female	3.78	4066.3	1.14	57.49	US Captive	February	Y	12420	387	Unrecorded						
92	Female		6347.6	1.26	42.03	US Captive	April	Y	10880	387	Basking						
92	Female		1795	0	0	US Captive	May	Y	10810	387	Resting in open						
92	Female		9832.2	0.88	134.1	US Captive	July	Y	10620	386	Resting in open						4.41
93	Sub-adult	1.74	5000			US Captive	July	N	1960	207	Resting in open	36.6	38.9			30	0.36
94	Sub-adult	0	16.4			US Captive	July	N	1980	204	Resting in open	36.6	44			30	2
94	Sub-adult	0	54.1			US Captive	June	N	3120		Unrecorded						
95	Sub-adult	0	307.3			US Captive	July	N	2220	227	Resting in vegetation	31.8	40.6			30	1.05
96	Sub-adult	0	10			US Captive	July	N	920	166	Resting in open	32.6	42.4			30	1.02
97	Sub-adult	0	91.7			US Captive	July	N	2050	206	Resting in open	32.7	43.6			30	1.07
98	Sub-adult	0	32.4			US Captive	July	N	920	159	Resting in open	31.8	40.6			30	0.49
99	Male	505.51	55.5			US Captive	July	Y	15620	470	Eating	25.9	26.1		0	40	1.21
99	Male	380.42	95			US Captive	December	Y	16250	448	Resting in vegetation	32	33.9				1.55
99	Male	789.05	272.6		0	US Captive	January	Y	17140	453	Reproductive/Nesting	24.3	26.7	38.3	40		
99	Male	1200	251.9			US Captive	February	Y	16340	450	Resting in vegetation	29.6	28.5	62.5			
99	Male	84.8	48.9			US Captive	October	Y	17610	450	Resting in vegetation	24.8	24.8	59.9	0	60	
99	Male	271.65	72.4			US Captive	November	Y	17130	450	Walking						
99	Male	135.78	41.2			US Captive	December	Y	17370	450	Resting in open						
99	Male	1200	114.5			US Captive	February	Y	17530	450	Unrecorded						
99	Male	1200	119			US Captive	April	Y	16650	450	Basking						
99	Male	1200	404.2			US Captive	May	Y	15610	450	Resting in open						
99	Male	1131.16	128.1			US Captive	June	Y	15730	450	Walking						3.1
99	Male	1200	467.4			US Captive	June	Y	15730	450	Unrecorded						
99	Male	1068.14	151.4		0	US Captive	July	Y	15750	447	Walking						2.55
100	Female		403.9	2.61	110.7	US Captive	July	Y	11360	382	Resting in open	30.2	30.2		0	60	0.42
100	Female		1126.5	2.31	41.54	US Captive	August	Y	10880	380	Resting in open	37.2	35.1	40	0	40	
100	Female		1109	2.29	145.74	US Captive	December	Y	10470	380	Resting in vegetation	32	33.1				1.12
100	Female		383.6	1.16	27.2	US Captive	January	Y	11000	384	Unrecorded	24.3	26.7	38.3	40		0.4
100	Female		1409	1.9	61.24	US Captive	February	Y	10630	380	Resting in open	29.6	28.5	62.5			0.36
100	Female		736	2.19	54.12	US Captive	September	Y	11540	380	Resting in open				0	30	
100	Female		1050.2	1.22	87.27	US Captive	October	Y	11510	380	Resting in vegetation	24.8	24.8	59.9	0	60	
100	Female		1107.4	1.29	72.73	US Captive	November	Y	10830	380	Resting in open						
100	Female		572.9	1.28	72.27	US Captive	December	Y	11250	380	Resting in open						
100	Female		1565.5	1.32	71.46	US Captive	February	Y	11650	380	Unrecorded						
100	Female		1578.5	0.62	58.76	US Captive	April	Y	11810	380	Resting in vegetation						
100	Female		556.4	0.86	87.81	US Captive	May	Y	12280	380	Walking						
100	Female		680.3	0.79	55.77	US Captive	June	Y	11440	380	Resting in vegetation						2.18
100	Female		636	0	169	US Captive	July	Y	10980	377	Resting in vegetation	27.4	23.7	47.9			2.42
101	Sub-adult	0	10		0	US Captive	July	N	2680	234	Resting in open	27.7	27.6		0	60	0.37
101	Sub-adult	0	28.1		0	US Captive	January	N	2820	239	Eating	24.3	26.7	38.3	30		1.2
101	Sub-adult	0	116.6		0	US Captive	February	N	2770		Resting in vegetation	29.6	28.5	62.5			0.46
101	Sub-adult	2.45	99.8			US Captive	November	N	3360		Walking						
101	Sub-adult	0	65.5		0	US Captive	December	N	3150		Resting in open						
101	Sub-adult	3.18	10			US Captive	June	N			Resting in open						2.04
101	Sub-adult	2.71	91.8			US Captive	July	N	3360	253	Basking	27.4	23.7	47.9			1.01
101	Sub-adult	2.61	774.3			US Captive	July	N	2090	219	Resting in open	27.4	23.7	47.9			3.58
102	Sub-adult	1.63	25.2			US Captive	July	N	1057	191	Resting in open	27.4	23.7	47.9			1.14

42	Female		2749.2	0.6	0	Madagascar Captive	March	N	8000	351	Unrecorded	27.1	26.7	80.5	0		
43	Sub-adult	0	317			Madagascar Captive	February	N	6200	311	Resting in open	30.9	30.4	82.4	70	60	0.36
44	Sub-adult	0	239.3			Madagascar Captive	February	N	5800	302	Resting in open	30.9	30.4	82.4	70	60	1.04
45	Sub-adult		2624.4			Madagascar Captive	January	N	2850	239	Resting in open				0	90	1
46	Sub-adult	0	99			Madagascar Captive	February	N	4700	277	Unrecorded	30.9	30.4	82.4	70	60	
47	Male	0	785.6			Madagascar Captive	May	N	7000	345	Eating	31.4	29.8	68.7	0	60	0.19
47	Male		921.8			Madagascar Captive	December	N	6600	352	Walking	26.7	27.3	89.5	100	40	0.22
47	Male	15.39	1149			Madagascar Captive	February	N	8000	367	Resting in open	30.8	31.6	77.7	70	70	0.56
47	Male	31.2	1245.4			Madagascar Captive	March	N	7400	366	Unrecorded	27.1	26.7	80.5	0		1.25
48	Sub-adult		5649.7			Madagascar Captive	May	N	4450	275	Resting in open	31.4	29.8	68.7	0	60	0.48
48	Sub-adult		3421			Madagascar Captive	December	N	4200	280	Resting in open	26.7	27.3	89.5	100	40	0.47
49	Sub-adult	0	600.7			Madagascar Captive	February	N	2550	228	Unrecorded	30.9	30.4	82.4	70	60	0.48
50	Sub-adult	0	249.1			Madagascar Captive	February	N	6000	300	Resting in open	30.9	30.4	82.4	70	60	
51	Sub-adult		1255.7			Madagascar Captive	January	N	4950	295	Resting in open				0	90	2.53
52	Female		570.8	0.93	36.49	Madagascar Captive	January	N	6200	320	Unrecorded						
52	Female		455.2			Madagascar Captive	December	N	6600	323	Walking	26.6	26.9	94.6	100	40	0.21
53	Sub-adult		26.4			Madagascar Captive	January	N	2650	238	Resting in open				0	90	1
54	Sub-adult		117.9			Madagascar Captive	May	N	2100	212	Eating	31.5	31.4	64.1	0	50	1.25
55	Sub-adult		37.5			Madagascar Captive	November	N	4400	282	Resting in open	35.6	35.4	53.2	70	90	0.23
56	Sub-adult		948.6			Madagascar Captive	January	N	3950	263	Walking				0	90	1.09
57	Sub-adult	3.11	4412.3			Madagascar Captive	May	N	4450	268	Eating	31.5	31.4	64.1	0	50	0.45
58	Sub-adult		262.5			Madagascar Captive	May	N	3650	256	Eating	31.5	31.4	64.1	0	50	1.24
59	Sub-adult		204.4			Madagascar Captive	May	N	4200	268	Eating	31.5	31.4	64.1	0	50	0.58
60	Sub-adult		1251.7			Madagascar Captive	January	N	3250	267	Resting in open				0	90	0.57
61	Sub-adult		121.7			Madagascar Captive	January	N	2550	234	Unrecorded						
62	Sub-adult		1722.1			Madagascar Captive	January	N	2350	228	Walking				0	90	0.36
63	Sub-adult		476.7			Madagascar Captive	January	N	2700	245	Walking				0	90	1.11
64	Sub-adult		2260.2			Madagascar Captive	January	N	3650	264	Resting in open				0	90	0.43
65	Sub-adult	7.13	579.3			Madagascar Captive	March	N	4500	291	Unrecorded	29.8	29	75.4	0		3.31
66	Sub-adult	0	1140.1			Madagascar Captive	March	N	4950	289	Unrecorded	29.8	29	75.4	0		0.3
67	Sub-adult	0	213.2			Madagascar Captive	March	N	4750	290	Unrecorded	29.8	29	75.4	0		1.24
68	Sub-adult		731.5			Madagascar Captive	November	N	3000	250	Walking	35.6	35.4	53.2	80	80	0.21
69	Sub-adult		144.1			Madagascar Captive	November	N	2400	234	Walking	35.6	35.4	53.2	70	80	0.26
70	Sub-adult		329.7			Madagascar Captive	November	N	4000	261	Unrecorded	35.6	35.4	53.2	80	80	0.25
71	Sub-adult		303.7			Madagascar Captive	November	N	3000	247	Walking	35.6	35.4	53.2	80	80	1.49
72	Sub-adult		338.5			Madagascar Captive	November	N	3000	244	Walking	35.6	35.4	53.2	70	80	0.37
73	Sub-adult		883.8			Madagascar Captive	November	N	2100	222	Walking	35.6	35.4	53.2	70	80	0.29
74	Sub-adult		239.4			Madagascar Captive	November	N	2800	250	Walking	35.6	35.4	53.2	70	80	0.3
75	Sub-adult		1004			Madagascar Captive	November	N	2600	239	Resting in open	35.6	35.4	53.2	70	80	0.35
